# Lignin–Sustainable Polymer for Mn(II) Biosorption from Aqueous Media

**DOI:** 10.3390/polym18121523

**Published:** 2026-06-18

**Authors:** Elena Ungureanu, Bogdan M. Tofanică, Maria E. Fortună, Ovidiu C. Ungureanu, Răzvan Rotaru, Valentin I. Popa

**Affiliations:** 1 “Ion Ionescu de la Brad” Iasi University of Life Sciences, 3 Mihail Sadoveanu Alley, 700490 Iasi, Romania; elena.ungureanu@iuls.ro; 2 Institute of Macromolecular Chemistry “Petru Poni”, 41A Grigore Ghica Voda Alley, 700487 Iasi, Romania; rotaru.razvan@icmpp.ro; 3 “Vasile Goldis” Western University of Arad, 94 the Boulevard of the Revolution, 310025 Arad, Romania; ungureanu.ovidiu@uvvg.ro; 4 “Gheorghe Asachi” Technical University of Iasi, 73 Prof. Dr. Docent Dimitrie Mangeron Blv., 700050 Iasi, Romania; vipopa@tuiasi.ro

**Keywords:** lignin, Sarkanda grass lignin, *Sorghum bicolor* L., biostability, Mn(II)

## Abstract

In the context of the circular bioeconomy and environmental protection trends, the efficient use of renewable resources has become a driving force for industry, and lignin represents precisely a renewable carbon resource, abundant in terrestrial biomass that could become a sustainable substitute for fossil resources, under conditions of full exploitation. This study systematically evaluates the biosorption of Manganese (Mn(II)) from aqueous media using unmodified *Tripidium bengalense* (Sarkanda grass) lignin. Under optimal operating conditions (adsorbent dosage of 5 g/L, pH 6.5, and 20 °C), a highly competitive experimental adsorption capacity of 12.52 mg/g was achieved. Kinetic studies revealed exceptionally rapid uptake rates, with thermodynamic equilibrium established within the first 30 min, fitting perfectly with the pseudo-second-order (Ho-McKay) model (R^2^ ≥ 0.9998). Equilibrium data were best described by the Freundlich isotherm (R^2^ ≥ 0.9886), confirming chemisorption via preferential inner-sphere complexation on a heterogeneous surface. Thermodynamic analysis verified that the process is spontaneous (ΔG ranging from −13.24 to −26.19 kJ/mol) and endothermic (ΔH from 11.21 to 14.83 kJ/mol). FTIR, SEM-EDX, and TG/DTG analyses confirmed successful Mn–O coordination involving phenolic hydroxyl and carboxylic groups. Furthermore, the lignin showed excellent recyclability, maintaining a retention efficiency over 70% (70.7–85.8%) after three desorption-resorption cycles using 1N HCl. Ecotoxicological validation via *Sorghum bicolor* L. germination tests confirmed the complete detoxification of the post-adsorption filtrates (up to 100% germination capacity), while the Mn(II)-loaded lignin completely suppressed seed germination (0%), proving secure metal immobilization. These findings establish raw Sarkanda grass lignin as an efficient, scalable, and ecologically sustainable biosorbent for heavy metal remediation.

## 1. Introduction

The numerous industrial, technological, medical, agricultural, and even domestic applications of heavy metals have led to their pervasive presence in nature, which has raised significant concerns regarding their toxicity, genotoxicity, and carcinogenicity to living systems. These metals are also non-biodegradable, easily accumulate in the environment, and infiltrate the food chain over time [1,2]. In such circumstances, the identification, examination, and elimination of these pollutants from ecosystems becomes imperative to alleviate the resulting biotic crises [3,4].

Manganese is a naturally occurring element that can be found in soil, rocks, and water. It exists in the form of Mn(II) [5] which exhibits high mobility. While manganese is a basic and essential nutrient for living organisms, overexposure to this element can cause dangerous and irreversible damage to ecosystems and human health [6]. This exposure is primarily attributable to industrial activity, wherein manganese is extensively utilized as an alloying agent in ferrous and non-ferrous alloys. The accumulation of electrolytic manganese residues resulting from these processes contributes to leachate infiltration, thereby transforming it into the predominant source of manganese contamination in soil [7]. Furthermore, mining activities that interfere with natural manganese, as well as its use in the manufacture of bricks, batteries, ceramics, glass, catalysts, aluminum cans, and electronic components, are actions that favor the appearance of a high level of toxicity with a direct impact on all living systems [8].

According to the World Health Organization (WHO), the manganese content in drinking water should not exceed 0.1 mg/L. Excessive accumulation in the central nervous system can trigger neurotoxicity, leading to manganism, a cerebral neurological disorder that induces behavioral, cognitive, and motor dysfunctions. Furthermore, there are cumulative effects with hematological, nephrotoxic, endocrine, or hepatotoxic diseases [6]. In plants, at a concentration of 150 mg/kg of dry weight, it has been observed to trigger oxidative stress, thereby inhibiting enzymatic activity and preventing chlorophyll biosynthesis and photosynthesis [9]. In animals, it has been demonstrated to produce neuronal, testicular, hepatic lesions, reproductive and immune system dysfunctions, pancreatitis, or lung diseases, at a concentration of approximately 1000 mg/kg [10].

As a type of heavy metal, manganese is challenging to metabolize naturally and will gradually migrate into the environment and accumulate in living beings through the food chain, which is difficult to control. Therefore, treatment against manganese contamination must be implemented without delay [11]. A promising technique for the elimination and segregation of noxious species, such as heavy metal ions, from aqueous systems, even at minimal concentrations, is biosorption [12,13]. The sustainability of biosorption is supported from several perspectives, including the following:

- Practical profitability: The use of biosorption does not require sophisticated equipment, which can be a significant cost savings for the user.
- Selectivity: Biosorption favors the targeted removal of specific pollutants without facilitating the formation of harmful by-products, which can have a positive impact on human health and the environment.
- Biocompatibility with the environment: Biosorption includes natural materials or biosorbents derived from waste as adsorption substrates, which can help to reduce environmental impact [14].

Despite these evident advantages, the scalability of adsorption is encumbered by limitations stemming from dependence on operational conditions, challenges in adsorbent regeneration, and even the rapid saturation of adsorption centers [15]. The selection of adsorbents is widely acknowledged as a pivotal factor in determining the efficacy of adsorption processes. The selection of adsorbents must be guided by several key criteria, including emergence, versatility, regenerability, abundance, cost-effectiveness, non-toxicity, and, most notably, an extended specific adsorption surface [13]. The scientific literature proposes the utilization of low-cost adsorbents derived from abundant and renewable sources as sustainable alternatives to conventional adsorbents, while promoting the principles of circular bioeconomy based on the “zero waste” concept [16].

In light of these considerations, lignin, the second most abundant polymer in nature, can be classified as a biosorbent that aligns with the principles of sustainability. Lignin is a renewable resource that is classified as biodegradable and ecologically sustainable agricultural waste. It possesses porous structures that are rich in active functional groups, which renders it conducive to sorption processes [17,18]. Indeed, lignin and lignin-based materials have witnessed substantial advancements in recent years, particularly with regard to their capacity to remove heavy metals and antibiotics [19]. The existing literature indicates that for single-metal pollution, lignin and derived materials can achieve efficient adsorption through surface complexation and ion exchange [20,21]. For combined pollution, synergistic adsorption-photocatalysis systems can simultaneously degrade antibiotics and immobilize heavy metals [21,22].

Conclusive evidence from numerous studies indicates that a comprehensive understanding and interpretation of adsorption isotherms is paramount for optimizing the adsorption mechanism pathways and designing high-performance systems with scalability potential [23,24]. Linear regression analysis is a statistical method that can select the most appropriate adsorption model. It can quantify the distribution of adsorbed substances and verify the hypothetical veracity of the adsorption isotherm model [21,23,25]. In instances where adsorption occurs within a single layer on homogeneous surfaces, the Langmuir isotherm is employed. Conversely, if the process transpires across multiple layers on heterogeneous surfaces, the Freundlich isotherm is utilized [23,24,25,26,27]. These isotherms represent the prevailing models through which information regarding the adsorption efficiency is obtained, taking into account the adsorbent mass, the initial concentration of the pollutant, the initial pH of the aqueous solution, and the duration of contact between the adsorbate and the adsorbent [21].

Adsorption isotherms are critical for comprehending the interplay between adsorbate and adsorbent, as well as for ascertaining the maximum adsorption capacity of the adsorbent. Conversely, adsorption kinetics furnishes pivotal insights concerning the rate at which the dissolved substance is adsorbed and the duration of the adsorbate interaction at the solid-liquid interface [28,29]. The first-order Lagergren and second-order Ho-McKay mathematical models, through their linearized equations, facilitate the calculation of important parameters for estimating the efficiency and behavior of adsorption (adsorption capacity, rate constants, adsorption rates, or intraparticle diffusion coefficients). These models are applied to a wide category of adsorption systems [18,21,30,31] through the use of slopes and intercepts derived from graphical representations.

In the context of replacing fossil-based resources and advancing the circular bioeconomy, the valorization of abundant, renewable, and currently underutilized lignocellulosic residues has become a research priority. The present study proposed the valorization of Sarkanda grass lignin, a soda lignin derived from *Tripidium bengalense* (formerly classified as *Saccharum bengalense*), a highly valued herbaceous biomass source in the nonwood pulp and paper industry. Unlike wood-derived lignins, Sarkanda grass lignin is a typical herbaceous lignin, belonging to the G-S-H type (Guaiacyl-Syringyl-p-Hydroxyphenyl). The structural characteristic of this material lies in its higher density of free carboxylic and phenolic hydroxyl groups, stemming from a significant content of hydroxycinnamic acids (e.g., p-coumaric and ferulic acids), which confer superior chelating potential for transition metal ions [21].

Despite the environmental urgency of Manganese (Mn(II)) remediation, the state-of-the-art literature has predominantly focused on the biosorption of classic heavy metals such as Lead, Copper, and Cadmium, leaving a conspicuous research gap regarding the specific affinity of lignin toward Mn(II). Consequently, the static adsorption of Mn(II) from aqueous media onto this specific herbaceous lignin substrate is systematically investigated here for the first time.

The study aimed to achieve a twofold objective: first, to characterize the adsorption mechanism through an integrated approach encompassing kinetics, isotherms, thermodynamics, and comprehensive structural elucidation via spectral, thermal, and surface analyses; and second, to demonstrate the material’s industrial viability by assessing the performance of the regenerated biosorbent over three consecutive desorption-resorption cycles. Furthermore, this work introduces a critical biological validation component by evaluating the biostability of the system through *Sorghum bicolor* L. germination tests.

This integrative approach confirmed not only the efficient retention of Mn(II) within the lignin matrix but also the ecological safety of the treated aqueous effluents. By establishing optimal operational parameters—including contact time, adsorbent dosage, and pH—this research positions Sarkanda grass lignin as a high-performance, economically viable, and ecologically sustainable alternative for heavy metal remediation, offering a robust framework for scalable ‘zero-waste’ environmental applications.

## 2. Materials and Methods

### 2.1. Materials

Chemical materials: Unmodified alkaline Sarkanda grass lignin supplied by Granit Récherche Development S.A., Lausanne, Switzerland [18], MnSO_4_ 5H_2_O and HCl 37% supplied by ChimReactiv S.R.L., Bucharest, Romania.

Biological material: *Sorghum bicolor* L. (var. Moench) seeds (humidity 11.8%, protein 12.0%, starch 70.8%, lipids 3.5%, fiber 7.8%, ash 1.7%) offered by “Ion Ionescu de la Brad”, Iasi University of Life Sciences, Iasi, Romania.

### 2.2. Experimental Procedure

#### 2.2.1. Adsorption Experiments

The adsorption process and its efficiency are contingent upon the phases involved (i.e., adsorbent and adsorbate) as well as a series of experimental parameters, including adsorbent dose, initial pH of the aqueous solution, adsorbate concentration, and contact time [32]. Consequently, it is imperative to conduct experimental tests in advance to establish the optimal conditions for optimizing the process [33]. To ensure an adequate number of active centers in the lignin structure for adsorption, a 5-g adsorbent mass was employed [21].

To establish the optimal conditions for the Mn(II) adsorption process, a series of preliminary batch experiments were conducted. The effect of the adsorbent dosage was evaluated in the range of 1–40 g/L, and the initial solution pH was varied from 0 to 8.0. These preliminary tests were essential to identify the point of maximum removal efficiency specific to the lignin matrix. Based on these optimization tests, a dosage of 5 g/L and an initial pH of 6.5 were selected as the optimal parameters for all subsequent kinetic, isotherm, and thermodynamic studies.

The functional groups present within the lignin structure have been demonstrated to engage in complexation reactions with heavy metal ions [19], thereby yielding stable ligno-complexes [18]. Consequently, the pH level emerges as a pivotal factor that can modulate the processes of protonation and deprotonation [3,21]. In strongly acidic and moderately acidic environments (below pH 5.5), manganese demonstrates high mobility and solubility, which hinders its removal from water. Conversely, in alkaline media (over pH 8), manganese becomes insoluble, readily oxidizable, and prone to the formation of precipitates [34]. This disrupts adsorption, which can be mitigated or even blocked [21,35,36]. In consideration of the aforementioned factors and the outcomes of the prelim inary tests, it was determined that pH 6.5 would be the optimal level. Preliminary monitoring of the pH at the end of the adsorption cycles confirmed that fluctuations were negligible (ΔpH < 0.1), thereby ensuring that the observed adsorption capacity reflects the conditions at the target pH.

The stock solution of Mn(II) in a concentration of 1000 mg/L was prepared by dissolving manganese (II) sulfate pentahydrate in distilled water. The working solutions were prepared by diluting with distilled water an exact volume measured from the stock solutions. The following solutions are prepared: 5.4938, 10.9876, 16.4814, 21.9752, 27.469, 32.9628, 38.4566, 43.9504, 49.4442, and 54.938 mg/L. A volume of 20 mL of solutions at the specified concentrations was added to each lignin sample, followed by a rest period at three different times: In order to ascertain the optimal duration for Mn(II) adsorption and to reach equilibrium, the following durations were investigated: 30, 60, and 90 min. These durations were investigated under laboratory conditions [21].

The regeneration of lignin from Sarkanda grass was investigated for its potential reuse. The primary objective of this study was to assess the adsorbent’s readsorption capacity. It was observed that the adsorbent, once utilized for water purification, often becomes a concentrated and toxic solid waste. This phenomenon poses a significant challenge to the life cycle of any sorption technology [18].

Subsequent to attaining adsorption equilibrium, Sarkanda grass lignin underwent regeneration with 1N HCl, and the efficiency of its absorption capacity was appraised following the execution of three desorption-resorption cycles.

25 mL of a 1N HCl solution and distilled water (1:1) mixture was added to the used lignin under magnetic stirring for 10 min, followed by simple filtration. The solid fraction was subjected to a drying process for a duration of 40 min at a temperature of 80 °C within an oven. Subsequent to this, an electronic analytical balance was employed to ascertain the precise mass, which was then utilized for the readsorption of the noxious species [21]. The experimental procedure was executed in strict accordance with the protocol established during the initial stage of adsorption, employing three replicates to ensure the reliability of the experimental results. The specified concentrations of MnSO_4_·5H_2_O were added to the regenerated lignin, and the mixture was allowed to rest for designated periods of time (30, 60, and 90 min). The isolation of the phases was conducted through a straightforward filtration process.

To summarize, the effect os key operating parameters was investigated as follows:

- Adsorbent Dosage: Ranging from 1 to 40 g/L (optimum established at 5 g/L).
- Initial pH: Ranging from 0 to 10 (optimum established at 6.5).
- Initial Mn(II) Concentration: Ranging from 5.49 to 54.94 mg/L.
- Contact Time: 30, 60, and 90 min.

The adsorption experiments were conducted in batch mode in a controlled laboratory environment, at a controlled temperature of 20 ± 0.1 °C. During the contact time, the mixture was subjected to periodic manual agitation to ensure the suspension of the lignin particles and to maintain homogeneous contact between the Mn(II) ions and the solid-liquid interface. All experiments were conducted in triplicate.

#### 2.2.2. Kinetic Studies, Isotherm and Thermodynamic Studies

To evaluate the adsorption kinetics, the Mn(II) uptake was measured at predetermined intervals (30, 60, and 90 min) using a constant initial concentration (5.49–54.94 mg/L) and an optimal dosage of 5 g/L and pH 6.5.

Equilibrium isotherms were generated by varying the initial Mn(II) concentrations (ranging from 5.49 to 54.94 mg/L) at a fixed adsorbent dosage of 5 g/L and pH 6.5, with a 60-min contact time. Thermodynamic parameters were determined by performing these equilibrium experiments at three distinct temperatures: 20 °C, 25 °C, and 30 °C, keeping all other parameters constant.

#### 2.2.3. Germination Experiments

From a biological standpoint, the efficiency of Sarkanda grass lignin in Mn(II) retention was evaluated through the design of germination tests on *Sorghum bicolor* L. (Moench) seeds. The experimental setup involved the parallel exposure of the seeds to two distinct phases obtained from the adsorption stage: the Mn(II)-loaded solid lignin matrix and the corresponding post-adsorption filtrates. For comparison, distilled water was utilized as the control for the liquid phase, while uncontaminated lignin served as the control for the solid phase. This biological exposure design was maintained over a seven-day vegetation period to monitor plant development [37].

### 2.3. Characterization Methods

#### 2.3.1. Spectrophotometric Determination of Mn(II)

The determination of Mn(II) concentration was achieved through the implementation of the potassium periodate method in an acidic medium, with the absorption spectrum collected at 525 nm [38]. Consequently, the quantitative determination of Mn(II) in the filtered solutions was performed using an accurately measured volume (2 mL) in accordance with the experimental procedure. The concentration value for each sample was calculated from the regression equation of the calibration curve. The spectrophotometric analysis was conducted using a laboratory-grade visible spectrophotometer (model VS−721N; wavelength range: 300–1000 nm; manufacturer: JKI, Shanghai, China).

#### 2.3.2. Isotherm Models

Adsorption isotherm models can provide information about the mechanism of the adsorption process, which is important for the design of the adsorption system [21,39]. In this context, the scientific literature proposes a series of analytical models to describe adsorption, such as: Temkin, Jovanovic, Elovich, Langmuir, and Freundlich each provide a distinct mathematical framework for interpreting how substances interact with surfaces. However, they differ in terms of assumptions and applicability [21,40].

The adsorption capacities were determined according to the following Equation [3]:

q = (c_i_ − c_e_)V/m, (mg/g) (1)

where c_i_ is the initial concentration (mg/L), c_e_ is the equilibrium concentration (mg/L), V is the volume of metal ion solution (L), m is the mass of adsorbent (g).

The most widely employed models for describing adsorption are the Langmuir and Freundlich models [21,39]. The Langmuir isotherm is predicated on the assumption that adsorption occurs on a homogeneous surface with a finite number of identical sites, each site containing a single molecule without interactions between adsorbed molecules, reaching saturation. In contrast, the Freundlich isotherm applies to heterogeneous surfaces with a non-uniform distribution of the heat of adsorption on the surface, allowing multilayer adsorption and interaction between adsorbed molecules [21,40,41].

The Langmuir Equation can be written in the following linear form [42]:

c_e_/q_e_ = 1/q_m_·k_L_ + c_e_/q_m_ (2)

where q_e_ is the amount of metal ions adsorbed per unit of mass of adsorbent (mg/g) at equilibrium, q_m_ is the maximum amount of metal ions retained on the absorbent after saturation (mg/g), K_L_ is the Langmuir constant (L/mg), c_e_ is the equilibrium concentration of metal ions in solution (mg/L).

The linear form of the Freundlich isotherm is as follows [43]:

log q_e_ = log k_F_ + 1/*n* log c_e_ (3)

where q_e_ is the amount of metal ions adsorbed per unit of mass of adsorbent (mg/g) at equilibrium, K_F_ is the Freundlich constant (mg/g) (L/mg)^1/*n*^, *n* is constant characterizing the affinity of metal ions to adsorbent, c_e_ is the concentration at equilibrium of metal ions in solution (mg/L).

The correlation coefficients (R^2^) were calculated using the least squares method, and their values indicate the most appropriate model for reproducing the experimental isotherm data [23].

#### 2.3.3. Thermodynamics of Adsorption

One of the fundamental requirements for characterizing and optimizing a process such as adsorption is the evaluation of the thermodynamic behavior, which provides useful indications regarding the performance and feasibility [44]. Therefore, with the assistance of the van’t Hoff and Gibbs laws, a series of thermodynamic parameters can be calculated (variation of free energy (ΔG), enthalpy (ΔH), and entropy (ΔS)), capable of providing predictable information regarding the spontaneity and energetic nature of adsorption. These aspects are of major importance in assessing the efficiency of the process and its sustainability [18,21,45].

#### 2.3.4. Kinetics Modeling

Kinetic adsorption models are instrumental in elucidating the rates and mechanisms by which adsorbates interact with adsorbents, thereby providing insights into the optimization and sustainability of adsorption processes [3,46,47]. The establishment of kinetic models for complex chemical systems, such as biomass utilization, poses significant challenges. This is due to the fact that the kinetic modeler must test different models and adjust several observables [21,46]. In this context, a series of kinetic models recommended in the literature (Lagergren, Ho-Mc Kay, Weber-Morris, Boyd, Elovich) were tested in the present study [47]. Through linear regression, the kinetic model that most faithfully described the experimental results was established [21,48]. Therefore, given the data obtained, the first-order Lagergren model and the second-order Ho-Mc Kay model were selected, as they are the most common analytical models employed for the monitoring of adsorption process kinetics [49].

The Lagergren model is applied to a liquid-solid adsorption system, being mathematically represented by the relation [50]:

ln [q_e_/(q_e_ − q)] = k_1_·t (4)

The mathematical equation of the Ho-McKay model reproduces the adsorption capacity of the solid adsorbent [50,51]:

t/q_t_ = (1/k_2_·q_e_^2^) + t/q_e_ (5)

where k_1_, k_2_ are constant adsorption rates for model 1 and 2, q_e_, q_t_ represents the adsorption capacity at equilibrium and at time t, respectively.

#### 2.3.5. Thermal Analysis

Thermostability is interdependent with adsorption kinetics, providing clear information on the adsorption capacity of a material by measuring mass changes as a function of temperature [52]. In this study, the thermal behavior of lignin was evaluated by performing thermogravimetric (TG) and differential thermogravimetric (DTG) analyses using the STA 449F1 Jupiter equipment (Netzsch Company, Selb, Germany). The thermal stability of the samples was assessed by measuring the percentage of mass loss from ambient temperature to 700 °C at a heating rate of 10 °C/min in an inert nitrogen atmosphere [21].

#### 2.3.6. Phytotoxicity Assessment

Following the exposure setup described in Section 2.2.3, the biological assessment of the *Sorghum bicolor* L. (var. Moench) seeds was characterized under controlled laboratory conditions (20 ± 1 °C, lighting regime of 15 h light/9 h dark). According to the established methodology [21,37], the seeds were initially subjected to a 5-min disinfection process using 5% sodium hypochlorite, followed by three washes with Milli-Q ultrapure water. Subsequently, the seeds were transferred into test tubes (180 × 18 mm) for an incubation period of one hour with intermittent shaking to facilitate soaking with the post-adsorption filtrates (ranging from 5.49 to 54.94 mg/L Mn(II)) or distilled water.

Following this, the seeds (10 per batch, in triplicate) were distributed uniformly in Petri dishes (90 × 15 mm) equipped with two overlapping filter paper discs [3,21]. The phytotoxicity of the loaded lignin and filtrates was quantitatively characterized by calculating the germination energy (E_g_) after three days and the germination faculty (F_g_) at the end of the 7-day period, excluding seeds exhibiting signs of rot or mold, as outlined in the literature [37]. The calculation equations are as follows [53]:

E_g_ = (a/*n*)·100 (%) (6)

F_g_ = (b/*n*)·100 (%) (7)

where a is the number of seeds germinated after three days, *n* is the total number of seeds analyzed, b is the number of seeds germinated at the end of the period (seven days).

#### 2.3.7. Morphological and Spectral Analyses

Scanning electron microscopy (SEM) is a highly utilized and effective instrumental method for examining and analyzing micro- and nanoparticles, as well as for imaging and characterizing solid objects [54]. It is frequently integrated with energy dispersive X-ray spectroscopy (EDX), an advanced microanalytical technique that can provide qualitative and semiquantitative information about the analyzed sample and its composition, facilitating elemental analysis and the determination of chemical configuration [55]. Consequently, the equipment utilized for conducting SEM-EDX analyses included a Quanta 200 scanning electron microscope (5 kV) equipped with an EDX elemental analysis system (Ametek, Berwyn, PA, USA). This apparatus was used in the Brno, Czech Republic facility.

For both uncontaminated and Mn(II) contaminated Sarkanda grass lignin, as well as for treated and untreated sorghum seeds with a polluting species, Fourier transform spectroscopy (FTIR) was utilized, given that this non-invasive and versatile method functions by analyzing the vibrational modes of molecules, thereby providing a unique spectral “fingerprint” that allows for the characterization and identification of chemical structures and the quality control of complex systems [56]. For this purpose, the Bruker Vertex 70 spectrometer (Billerica, MA, USA) at a resolution of 2 cm^−1^ was employed.

#### 2.3.8. Desorption-Resorption Tests

The desorption of Mn(II) from used lignin was performed to examine the possibility of reusability of the adsorbent, so that it could be included in the sphere of sustainability and circulation. Following the isolation of the samples at the three designated experimental contact times, the filtrate obtained was utilized for the quantitative determination of the metal ion. This determination was made by employing a volume of 2 mL (in accordance with the stipulated experimental procedure) for each sample. The concentration of the contaminating species was calculated using the regression equation of the calibration curve. The desorption-resorption cycle was repeated thrice to obtain a relevant conclusion regarding the adsorption efficiency of the regenerated lignin.

The retention percentage was calculated, using the relationship [57]:

R = [(c_i_ − c_r_)/c_i_]·100 (%) (8)

where c_i_ is the initial concentration (mg/L), c_r_ is is the residual concentration (mg/L) of the metal ion.

#### 2.3.9. Statistical Analysis

To ensure the reproducibility of the results, the experimental procedures were performed in triplicate, and the data analysis was performed with Microsoft Excel (Office Professional Plus 2013, Microsoft Corporation, Redmond, WA, USA). The results were presented as mean ± standard deviation, thus ensuring the quantification of data variability [21].

## 3. Results and Discussion

### 3.1. Evaluation of the Adsorption Capacity of Sarkanda Grass Lignin for Mn(II) from Aqueous Media by Optimizing Experimental Conditions

#### 3.1.1. Sarkanda Grass Lignin Dosage

**Figure 1:**
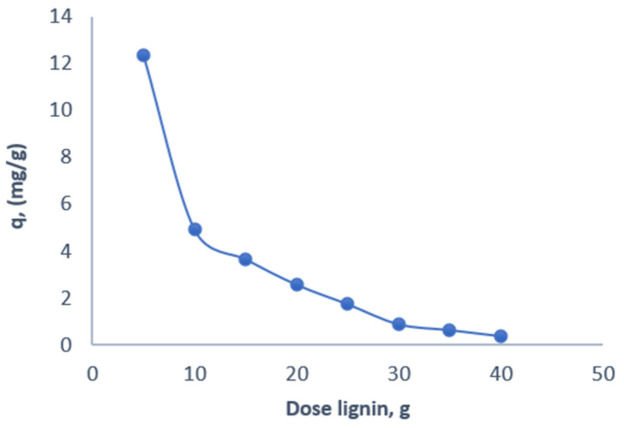
The influence of Sarkanda grass lignin dose on the efficiency of the Mn(II) adsorption in concentration 54.938 mg/L, contact time 60 min, pH 6.5.

The adsorbent dose exerts a significant influence on the dynamics and efficiency of adsorption, operating through the availability of active binding centers on its surface that engage in adsorbate retention at a given concentration [3,18,57]. Given the significance of this parameter, the establishment of an optimal dose for adsorption efficiency necessitates experimental evaluation. The scientific literature provides a range of 4–40 g/L for lignin [21,36,58]. Preliminary tests performed within this interval identified 5 g/L as the most suitable dose, providing a balance between efficient Mn(II) removal and effective utilization of lignin derived from Sarkanda grass (Figure 1).

As illustrated in Figure 1, which presents the dynamics of Mn(II) adsorption from aqueous solutions at a concentration of 54.938 mg/L at the contact time considered optimal of 60 min, it is evident that a substantial quantity of Sarkanda grass lignin does not result in a significant adsorption of Mn(II). Instead, there is a continuous decline in the amount of Mn(II) retained per unit mass of lignin, a phenomenon also documented in other studies [18]. Specifically, when 5 g/L of lignin is used, a capacity of 12.3601 mg/g is obtained; at 10 g/L, it drops to 4.9241 mg/g; and at an extreme dosage of 40 g/L, only 0.3822 mg/g is recorded.

This significant decline in specific adsorption capacity at high solid concentrations is not a result of pore saturation, but is rather governed by well-documented physical limitations in mass transfer [58,59]. At elevated adsorbent doses, the highly amorphous lignin particles tend to agglomerate and aggregate in the aqueous medium. This physical overlapping effectively reduces the total available macroscopic surface area, sterically blocking a significant portion of the active binding sites (such as the free carboxyl and phenolic groups) [21]. Furthermore, particle aggregation increases the diffusion path length, preventing the Mn(II) ions from fully accessing the internal active sites to form the robust manganese lignocomplexes typically generated by the strong affinity between the phases [36]. Concurrently, the excessive surplus of active sites relative to the fixed Mn(II) concentration leads to a mathematically lower uptake per unit mass. Consequently, the 5 g/L dosage was identified as the optimal threshold, providing the ideal balance between available surface area and complete utilization of the active binding sites.”

#### 3.1.2. Initial Concentration of Mn(II)

**Figure 2:**
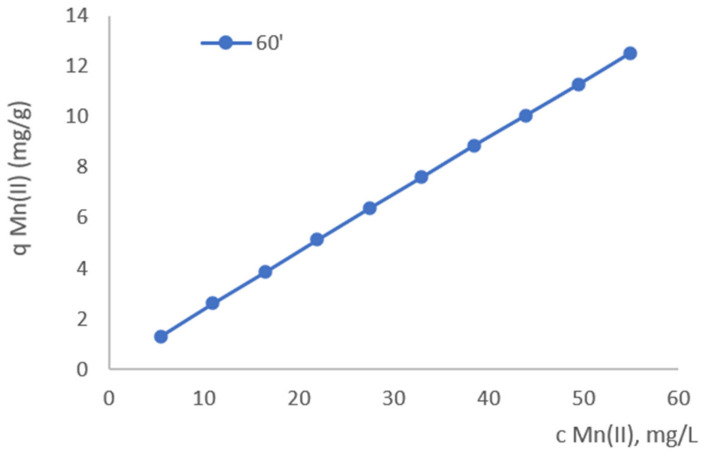
The adsorption capacity of the Sarkanda grass lignin, pH of 6.5.

The initial concentration of the metal ion is a critical factor in determining the effectiveness of biosorption, as it functions as a driving force to overcome the resistances to mass transfer between the aqueous and solid phases [60]. Furthermore, an increase in the initial concentration of the metal ion has been demonstrated to enhance the number of collisions between the metal ion and the biosorbent, thereby increasing the absorption of the metal species [21,60,61]. As illustrated in Figure 2, the adsorption capacity of Sarkanda grass lignin for Mn(II) from an aqueous medium at a contact time of 60 min demonstrates a direct proportionality between the two parameters. Therefore, an increase in the adsorption capacity of Sarkanda grass lignin is documented, from 1.3076 mg/g at a concentration of 5.4938 mg/L to 12.5187 mg/g at a concentration of 54.938 mg/L. This finding suggests that an increase in the concentration of manganese(II) in the analyzed concentration range is associated with an increase in the adsorption capacity of Sarkanda grass lignin. This phenomenon can be explained by considering the propensity of lignin to complex with metal ions following activated adsorption [21,62].

#### 3.1.3. Contact Time Between Phases

**Figure 3:**
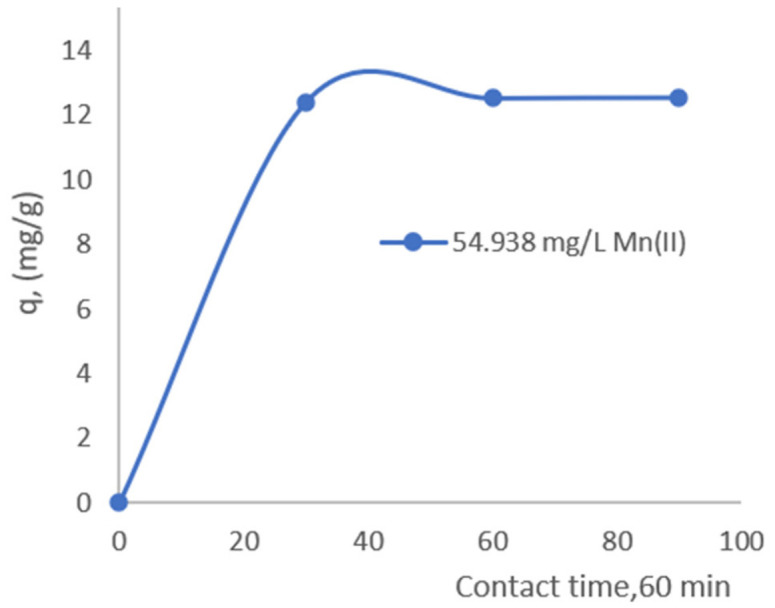
The influence of contact time on the adsorption of Mn(II) on Sarkanda grass lignin, pH 6.5.

The determination of the contact time is crucial for ascertaining the optimal duration for the adsorption of heavy metals, a factor that directly impacts the application sphere. This is due to the interdependence of the process with the kinetics and the chemical equilibrium between the adsorbent and the adsorbate [63]. To this end, the adsorption capacity of Sarkanda grass lignin for Mn(II) was analyzed for all ten initial concentrations (ranging from 5.49 to 54.94 mg/L) at three distinct intervals: 30, 60, and 90 min. The results consistently indicated that the adsorption capacity increases until 60 min, after which the system reaches phase equilibrium with no statistically significant increase in uptake. Subsequent to this, the adsorption proceeds slowly. For instance, Figure 3 illustrates the variation of the adsorption capacity (mg/g) at the three experimental times for the Mn(II) solution with a concentration of 54,938 mg/L. Consequently, at 30 min, q = 12.4037 mg/g is recorded, at 60 min, q = 12.5188 mg/g is recorded, and at 90 min, q = 12.5383 mg/g is recorded, which show that the differences between these values are statistically insignificant (*p* > 0.05). This indicates that the adsorption process is characterized by extremely rapid kinetics, with equilibrium effectively reached within the first 30 min of contact. This rapid equilibration is a key operational advantage, suggesting that the required contact time for industrial Mn(II) removal processes can be significantly shorter than initially assumed, thereby enhancing the throughput capacity of the biosorption system. This suggests that the 60-min time point is most favorable under the established experimental conditions (Figure 3).

#### 3.1.4. pH Initial Solution

**Figure 4:**
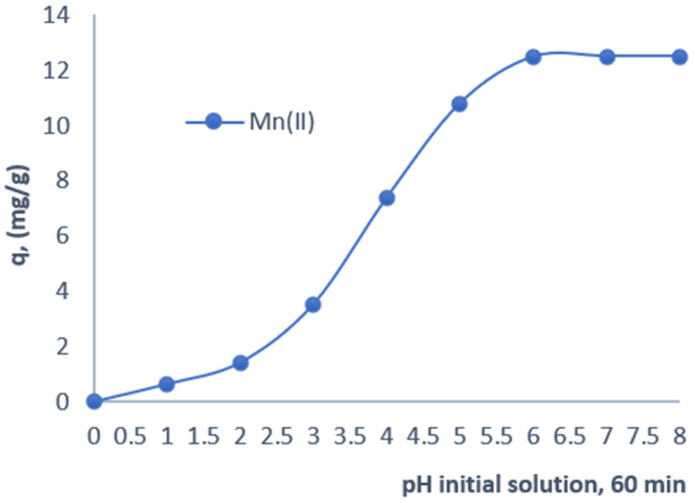
The influence of the pH of the initial solution on Mn(II) adsorption on Sarkanda grass lignin.

The initial pH of the solution is a critical factor in the adsorption of metal ions in adsorbents, as it affects the surface charge of the adsorbent, the degree of ionization, and the specifications of the adsorbate [64]. It is well established that in acidic media (pH ˂ 5.5), the solubility and mobility of manganese are high, which hinders its removal from aqueous media [34,62]. Additionally, the excess of protons can compete with manganese ions for binding sites on lignin [3]. Conversely, in alkaline media (pH ˃ 8), the insolubility of manganese is high, favoring precipitation [34,62], which can impede adsorption [21]. Furthermore, lignin demonstrates a notable adsorption capacity within the pH range of 4.0 to 6.5 [45]. Consequently, preliminary tests were conducted to ascertain the variation of the adsorption capacity of Sarkanda grass lignin depending on the initial pH of the aqueous Mn(II) solution, with the pH range of the solution being from 0 to 8. As demonstrated in Figure 4, the adsorption capacity of lignin exhibits a direct proportional relationship with the increase in the pH of the Mn(II) solution. This increase in pH has been shown to enhance the probability of functional groups, particularly carboxyl and hydroxyl groups, within lignin, engaging in bond formation with the metal ion [36]. The maximum adsorption capacity of 12.48 mg/g was attained at a pH of 6.5, which is regarded as the optimal pH for this process.

To fundamentally understand the dependence of adsorption capacity on the initial pH, the surface charge of the adsorbent must be considered in relation to its point of zero charge (pH_pzc_) [65]. Although not experimentally determined in the present study, the literature extensively documents that technical and herbaceous lignins typically exhibit a pH_pzc_ ranging from 3.5 to 4.5 [66]. This is primarily governed by the dissociation of carboxylic acid groups present in the lignin structure [67]. When the solution pH is higher than the pH_pzc_ of the adsorbent (pH > pH_pzc_), the surface functional groups—specifically the carboxylic and some phenolic hydroxyl groups—undergo extensive deprotonation. At the experimentally determined optimal pH of 6.5, the surface of the Sarkanda grass lignin acquires a strong net negative charge. This negative surface potential significantly reduces electrostatic repulsion and maximizes the powerful electrostatic attraction toward the positively charged Mn^2+^ ions. Conversely, at highly acidic pH values (pH_pzc_ < pH_pzc_), the lignin surface is predominantly protonated and positively charged, creating a strong electrostatic repulsion that competes with Mn^2+^ for the active binding sites, thereby inhibiting adsorption.

Thus, reaching pH 6.5 ensures the ideal thermodynamic environment and the maximum adsorption capacity is attained. Prior experiments confirmed that bulk precipitation of Mn(II) does not occur at this pH in the absence of the adsorbent, ensuring that the removal is strictly adsorption-driven. The high retention capacity observed can be attributed to the optimal deprotonation of the oxygen-containing functional groups within the Sarkanda grass lignin, particularly the carboxylic (pKa ~4–5), phenolic hydroxyl groups and methoxy groups [68]. At this pH, the negatively charged lignin matrix minimizes electrostatic repulsion and acts as a powerful chelating framework [69]. The Mn^2+^ ions are strongly attracted to the surface, where they form stable inner-sphere complexes via Mn–O coordination interactions with the electron-rich oxygen atoms of the lignin structure, as suggested by the shifts in the O–H and C–O vibrational bands observed in the FTIR spectra. Thus, the enhanced removal efficiency is governed by the development of the electrostatic attraction to heavy metal ions by lignin, and the complexation to heavy metal ions by lignin, rather than precipitation [70].

### 3.2. Modeling of Adsorption Equilibrium of Mn(II) onto Sarkanda Grass Lignin

Adsorption isotherm models can provide information about the mechanism of the adsorption process, which is important for the design of the adsorption system [39]. Langmuir-Freundlich isotherms have been established by their contribution to establishing the adsorption equilibrium, which is particularly useful for the practical sphere [21,71]. These isotherms include two types of adsorption processes. The first type is the development of adsorption on a single layer on an adsorbent with a homogeneous surface and a limited number of active centers (Langmuir isotherm) [72]. The second type is the development of adsorption in a single or multiple layers on an adsorbent with a heterogeneous surface and chaotic distribution of adsorption energy on the specific surface (linear Freundlich isotherm) [73]. In accordance with the linear representation of the regression equation of each model, the correlation coefficients (R^2^) are calculated. The adsorption performance is then evaluated based on the values of these coefficients [25]. Consequently, the magnitude of the correlation coefficients (R^2^) is indicative of the efficacy of the applied model in characterizing the adsorption process [74].

**Figure 5:**
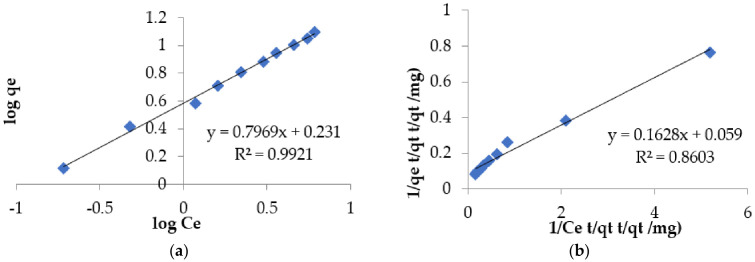
Freundlich adsorption model (**a**) and Langmuir adsorption model (**b**) for Mn(II) adsorption onto Sarkanda grass lignin, contact time 60 min.

As illustrated in Figure 5a,b, the Freundlich and Langmuir models are employed to elucidate the adsorption of Mn(II) from aqueous media on Sarkanda Grass lignin under working conditions that have been established as optimal. The experimental conditions included a temperature of 20 ± 0.1 °C, a contact time between phases of 60 min, and a pH of 6.5. The specific indices of the Freundlich (R^2^, 1/n, k_F_) and Langmuir (R^2^, q_m_, K_L_) models are presented in Table 1 across the three contact times (30, 60, and 90 min). It is important to clarify that by strict thermodynamic definition, adsorption isotherms must be evaluated only once the system has stabilized. As established by our kinetic analysis, the interaction between Mn(II) and the Sarkanda grass lignin is exceptionally rapid, with adsorbtion equilibrium being effectively attained within the first 30 min. Therefore, the data evaluated at 30, 60, and 90 min do not represent transient kinetic stages, but rather the maintained state of thermodynamic equilibrium over time. The evaluation of the Langmuir and Freundlich models across these consecutive intervals serves as an empirical diagnostic validation. The stability of the calculated parameters confirms that a robust and stable thermodynamic equilibrium has been achieved and maintained.

As illustrated in Table 1, the correlation coefficients (R^2^) derived from the Langmuir model are relatively low, ranging from 0.7536 to 0.9091, whereas those obtained for the Freundlich model exhibit a vastly superior fit, falling between 0.9886 and 0.9921. This stark contrast is not merely a statistical deviation but a fundamental physical reflection of the lignin’s surface chemistry. The Langmuir isotherm strictly assumes a perfectly homogeneous surface with uniform binding energies and strict monolayer coverage. However, *Tripidium bengalense* lignin is a highly amorphous macromolecule possessing a strongly heterogeneous surface with diverse functional groups (carboxylic, phenolic, and aliphatic hydroxyls) that exhibit varying affinities and binding energies for Mn^2+^. Consequently, the system deviates significantly from Langmuir assumptions. This is further supported by the low Langmuir constants *K_L_* (0.0804 → 0.0815), which indicate that the surface is heterogeneous and that adsorption likely extends beyond a single layer.

Instead, the experimental data provide robust validation for the Freundlich model, which accurately accounts for this surface heterogeneity and complex chemisorption interactions. The obtained Freundlich constants, specifically the values for *K_F_* (1.8953 → 2.2188) and 1/n (0.9002 → 0.9973), indicate the presence of variable binding energies, pointing toward the occurrence of ion exchange interactions and superficial chelation [36].

It should be noted that the Freundlich parameters (*K_F_* and 1/*n*) exhibit slight variations between 30, 60, and 90 min. Unlike ideal synthetic adsorbents, raw lignin is a highly amorphous and heterogeneous biopolymer. These temporal fluctuations do not negate the achievement of macro-kinetic saturation; rather, they reflect the ongoing dynamic restructuring of the lignin matrix, internal swelling, and the progressive activation of deeper, higher-energy binding sites. This non-ideal behavior underscores the complexity of heavy metal chelation within natural macromolecules.

Together, these findings robustly corroborate the proposed mechanism of preferential inner-sphere complexation on a heterogeneous biopolymer matrix. Nevertheless, while the isotherm models strongly suggest a chemically driven pathway, they do not definitively elucidate the complete physical or chemical nature of the Mn(II) adsorption on Sarkanda grass lignin. A comprehensive understanding of the process spontaneity and reaction rates requires further validation from a thermokinetic perspective.

### 3.3. Thermodynamics of Mn(II) Adsorption on Sarkanda Grass Lignin

The equilibrium of adsorption is determined by thermodynamic factors, with a particular emphasis on the removal of heavy metals from wastewater. Consequently, a thermodynamically optimized adsorption system can provide sustainable solutions for the treatment of contaminated aqueous media [75]. The spontaneous and practical nature of the adsorption processes is evaluated using the Gibbs free energy and the compensating effects of enthalpy and entropy variations with temperature [21,76]. To this end, the thermodynamic behavior was evaluated at three distinct temperatures: 20 °C (293.15 K), 25 °C (298.15 K), and 30 °C (303.15 K), as summarized in Table 2.

As illustrated in Table 2, the Gibbs free energy (ΔG) exhibits a range of negative values from −26.19 to −13.24 kJ/mol across the tested temperature range. This observation indicates that the adsorption of Mn(II) on lignin occurs spontaneously and is thermodynamically favorable, following a mechanism that is predominantly governed by electrostatic interactions [76]. Furthermore, the values of ΔG become systematically more negative with an increase in temperature, demonstrating that higher thermal energy promotes and enhances the spontaneity of the adsorption process.

Similarly, the observed temporal shifts in the Gibbs free energy (ΔG) across the 30, 60, and 90-min intervals at constant temperatures are characteristic of complex biological matrices. Following the rapid initial macroscopic saturation, the internal lignin structure undergoes continuous conformational adjustments. The shifting ΔG values reflect the ongoing endothermic desolvation of deep-pore binding sites and the delayed rearrangement of the hydration shells surrounding the newly formed Mn(II)-lignocomplexes.

Regardless of the pH, positive enthalpy variation (ΔH) values ranging from 11.21 to 14.83 are observed. These values provide substantial evidence to support the hypothesis that adsorption exhibits an endothermic nature [71]. Consequently, an increase in temperature, within the limits of lignin thermostability, appears to promote the adsorption of Mn(II) on lignin. This phenomenon is accompanied by an intensification of molecular agitation, diffusion, and activation energy, which occurs concurrently with the dehydration of Mn(II) and the lignin surface [77].

In addition, the entropy variation (ΔS) has been observed to exhibit positive values within the range of 85.24 to 129.96 (see Table 2). These findings suggest a propensity for the adsorbate-adsorbent system to undergo an increase in disorder, which occurs as the adsorbate molecules surrounding the free groups of the adsorbent diminish their degree of ordering [21]. Furthermore, the data obtained in this study are consistent with those previously reported in the literature for established adsorbents [78]. Additionally, the findings are reproducible and align with the deductions derived from the analysis of adsorption isotherms.

### 3.4. Kinetic Modeling of the Adsorption of Mn(II) onto Sarkanda Grass Lignin

Adsorption kinetics provides valuable information about possible adsorption mechanisms and their potential rate-limiting step during the process [21]. The rate of adsorption is contingent upon the number of particles adsorbed on the adsorbent surface per unit time and the number of particles colliding per unit surface per unit time [79]. In essence, kinetics is defined as the measure of adsorption efficiency, offering evidentiary support for the adsorption rate and the contact time of the adsorbates at the solid-liquid interface. This parameter is a critical criterion in the selection of optimized process parameters [80].

The kinetic models frequently employed to describe liquid-phase adsorption on solid supports and to assess the adsorption performance of the adsorbent include the pseudo−1st order Lagergren model and the pseudo−2nd order Ho-McKay model [74], depending on the level of error deduced from the value of the correlation coefficient (R^2^), opting for the most appropriate model [21,79].

**Figure 6:**
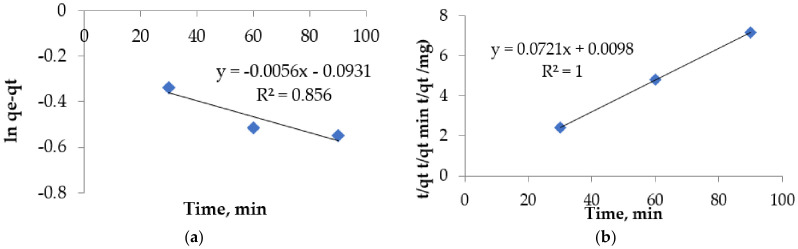
Linear representation of the Lagergren pseudo-I order model (**a**) and Ho-McKay pseudo-II order model (**b**) for adsorption of Mn(II) onto Sarkanda grass lignin, contact time 60 min.

The linear dependence for the two kinetic models applied to evaluate the adsorption of Mn(II) from aqueous media onto Sarkanda grass lignin, at the initial normalized concentration of 100 mg/L, is reproduced in Figure 6a,b. In this case, the first-order Lagergren model and the second-order Ho-McKay model are employed. Table 3 presents the specific kinetic indices, calculated from the slopes and the intercept with the ordinate of the linear dependences obtained for the two models. The correlation coefficients (R^2^) were obtained by linear regression [21].

As illustrated in Table 3, the pseudo-first-order Lagergren model yields relatively low correlation coefficients (R^2^) ranging from 0.5978 to 0.8516. Because of this inadequate mathematical fit, the physical parameters derived from the Lagergren model (k_1_ and q_e_) cannot be reliably used to interpret the kinetic mechanism of the system. Nevertheless, presenting this model’s limitation is highly valuable; the statistical inadequacy of the pseudo-first-order model—which typically describes physical adsorption—empirically demonstrates that physical forces are not the dominant pathway in this biosorption process.

Conversely, the pseudo-second-order Ho-McKay model exhibits a diametral mathematical fit, with correlation coefficients (R^2^) ranging from 0.9998 to 1.0000 (approaching unity in all cases, as shown in Table 3). This outstanding agreement establishes the Ho-McKay model as the ideal mathematical representation for evaluating the kinetics of Mn(II) adsorption on Sarkanda grass lignin. This successful fit, further substantiated by the physically consistent values of the calculated adsorption capacity (q_e_) and the rate constant (k_2_), strongly confirms that the rate-controlling step of the adsorption process is governed by chemisorption (surface chelation) involving the active functional groups of the lignin matrix.

Therefore, the Ho-McKay model posits that the kinetics of Mn(II) adsorption on Sarkanda grass lignin are driven by the chemical interaction between Mn(II) and the functional groups in the lignin structure. This interaction denotes the existence of a chemosorption process that can generate changes in the internal structure of lignin by cleaving and forming new bonds, most likely dative. This process favors the formation of relatively stable lignocomplexes as a result of the good affinity between lignin and Mn(II) [62]. These lignocomplexes precisely demonstrate that the rate-controlling step of the entire removal process is chemisorption, thereby validating the excellent mathematical fit and the unity correlation coefficients (R^2^ ≈ 1) obtained with the pseudo-second-order kinetic model.

It is critical to acknowledge a methodological limitation regarding the time-dependent evaluation of this system. From a strict physicochemical perspective, conducting a comprehensive transient kinetic study requires a high density of early-stage contact times (e.g., under 20 min) to capture the rapid initial uptake phase. In our study, the evaluation at 30, 60, and 90 min exclusively captured the thermodynamic plateau, as the actual rapid adsorption phase concluded well before the 30-min mark due to the high chemical affinity of the lignin.

Consequently, the application of classical kinetic models (such as the pseudo-second-order model) to these three specific data points does not serve as a fundamental evaluation of transient diffusion rates. Instead, the resulting excellent mathematical fit (R^2^ ≈ 1) acts as a macro-kinetic indicator, empirically confirming that the chemisorption process had already reached a highly stable and irreversible saturation plateau by 30 min. Future scale-up studies must incorporate continuous flow or rapid-sampling batch setups to accurately map the initial mass transfer kinetics.

Regarding the comparative data, the calculated adsorption capacities (q_e,cal_) derived from the Ho-McKay kinetic model (please see Table 3) exhibit slight numerical deviations from the experimental adsorption capacities (q_e,exp_) reported in the isotherm studies (please see Table 1). These discrepancies primarily arise from the distinct hydrodynamic conditions and mass-transfer regimes between the two sets of experiments. The isotherm data in Tabel 1 represent the ultimate thermodynamic capacity attained under optimized, stable equilibrium conditions, whereas the kinetic parameters in Table 3 reflect the apparent adsorption behavior observed during the time-dependent experimental setup, which involved periodic manual agitation. As previously discussed, this mixing regime introduces a degree of mass-transfer resistance compared to the static equilibrium conditions used for isotherm construction, resulting in slightly lower apparent kinetic capacities. This variation is physically consistent with the non-ideal, heterogeneous nature of the lignocellulosic matrix and does not undermine the validity of the determined rate-controlling mechanism.

### 3.5. Evaluation of Mn(II) Adsorption Capacity from Aqueous Media by TG/DTG Thermogravimetric Analyses

**Figure 7:**
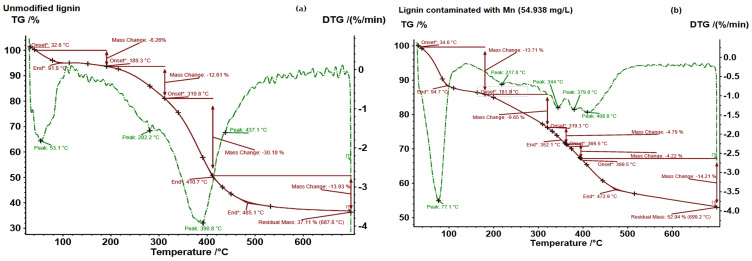
Thermogravimetric (TG, red) and derivative thermogravimetric (DTG, green) curves of Sarkanda grass lignin before adsorption (**a**) and after Mn(II) adsorption at 54.938 mg/L and 60 min contact time (**b**).

The thermal behavior of the lignin, as measured by TG and DTG analyses, provides a valuable metric for characterizing its adsorption properties as a function of temperature. This analysis facilitates the identification of weight losses and, by extension, specific desorption stages, such as the separation of free water from the bound adsorbate [52,81]. Consequently, the thermal analysis was conducted by estimating the percentage mass losses from ambient temperature to 700 °C [21], both for native lignin and for lignin samples loaded with aqueous Mn(II) solutions for the studied concentration range. As illustrated in Figure 7a,b, the TG and DTG curves were recorded for uncontaminated lignin and lignin contaminated with an aqueous Mn(II) solution at a concentration of 54.938 mg/L. The contact time for this experiment was set at 60 min, and the atmosphere used was nitrogen. As illustrated in Figure 7a, the mass losses of uncontaminated lignin exhibit maximum decomposition peaks at the following temperatures: The values obtained were 53.1, 282.2, 390.8, and 437.1 °C. Consequently, mass losses were documented at 91.8 °C (loss of 6.26%), 410.7 °C (loss of 30.18%), and 485.1 °C (loss of 13.93%), with a residual mass of 37.11% remaining at 687.8 °C. In the case of lignin treated with an aqueous solution of Mn(II) at a concentration of 54,938 mg/L, an contact time of 60 min, as illustrated in Figure 7b, the maximum decomposition peaks emerge at 77.1, 217.8, 344, 379.8, and 408.8 °C, and the weight loss occurs at 94.7 °C (loss of 13.71%), at 352.1 °C (loss of 4.79%), at 472.9 °C (loss of 14.21%), and at 699.2 °C, with a residual mass of 52.94%.

The findings of TG/DTG analysis suggest that the thermal stability of lignin is approximately 189.3 °C (uncontaminated lignin) and 181.9 °C (contaminated lignin), respectively. The subsequent stages are attributed to its decomposition. The initial decomposition process at a low thermal regime is associated with water loss, followed by the decomposition of systems and the loss of structural water. The weight loss in the residual mass domain corresponds to the thermal decomposition profile of the lignin fractions. The thermostability of lignin is associated with its structural-functional characteristics (the ratio of syringyl/guaiacyl, respectively the carbonyl, aromatic, and aliphatic hydroxyl groups) [21]. The difference in the remaining mass residues (from 37.11% for unmodified lignin to 52.94% for contaminated lignin) demonstrates the adsorption of Mn(II) in the pores of Sarkanda grass lignin.

### 3.6. Assessment of Mn(II) Adsorption Efficiency on Sarkanda Grass Lignin Through Biological Indices

#### 3.6.1. Number of Germinated *Sorghumb bicolor* L., Moench Variety

**Figure 8:**
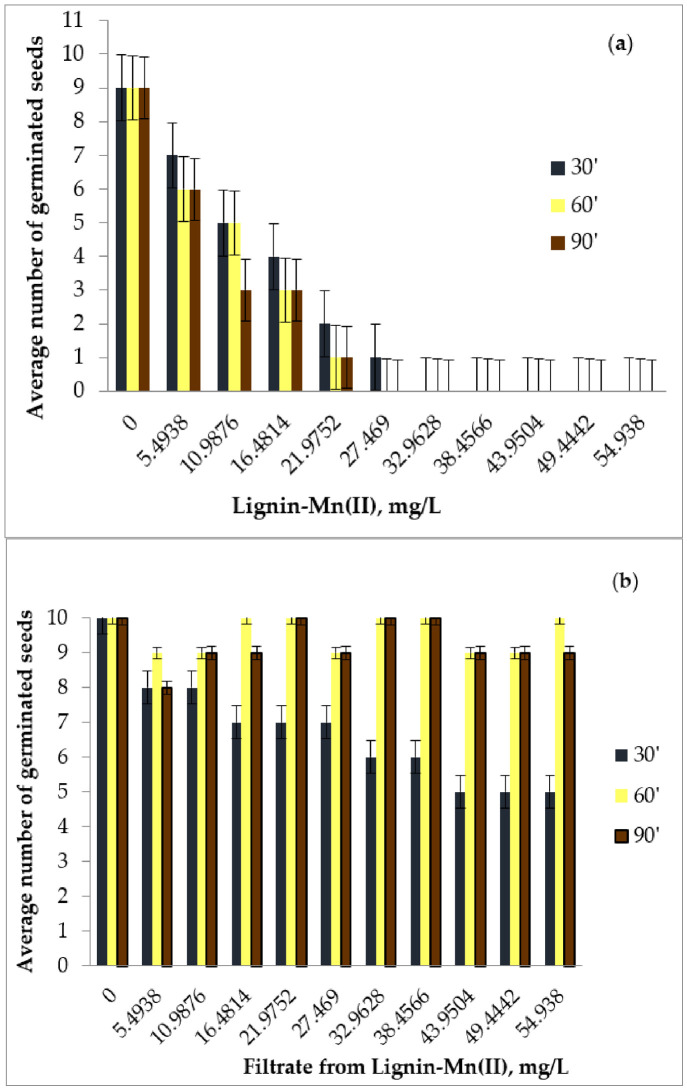

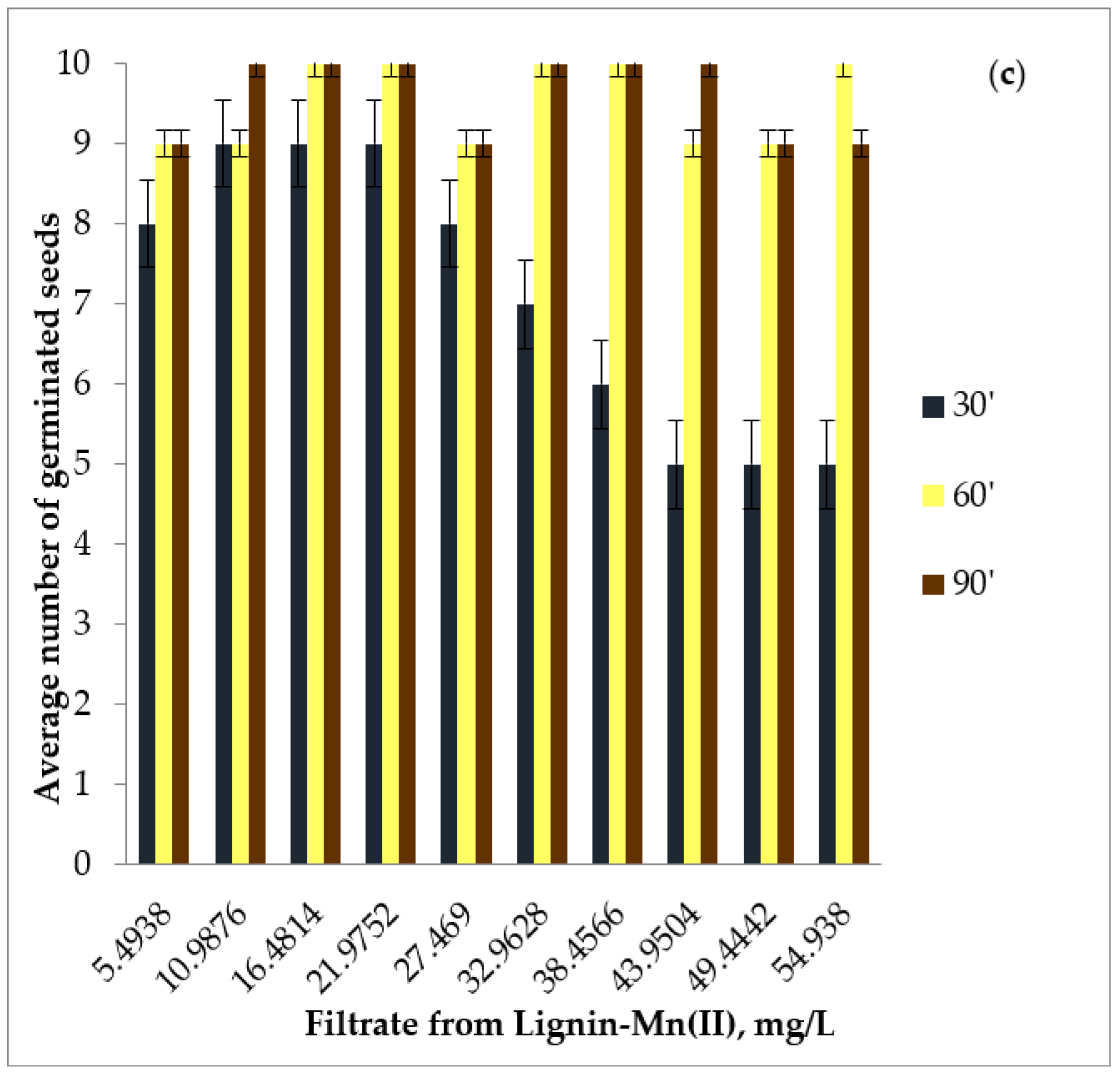
The average number of sorghum seeds germinated at 3 days for the contaminated samples (**a**) and for the filtrates resulting from Mn(II) adsorption at 3 days (**b**) and 7 days (**c**).

The presence of Mn(II) from contaminated lignin samples has the potential to disrupt the osmotic and physiological balance of plants, which is why germination tests were performed on sorghum seeds over a period of seven days, according to literature recommendations [82]. As illustrated in Figure 8a–c, the mean number of sorghum seeds that germinated was determined after three replicates at three days for samples of Sarkanda grass lignin contaminated with Mn(II) aqueous solution within the studied concentration range. Similarly, the mean number of seeds that germinated after three replicates at three and seven days for the filtrates obtained after Mn(II) adsorption at the three contact times was determined. The experimental data demonstrate an intensification of abiotic stress as the concentration of the metal ion and the contact time increase, thus accentuating the inhibitory effect of Mn(II) on sorghum seed germination.

In a study of the germination of 10 seeds from the control samples, it was found that the average percentage of germination for Sarkanda Grass lignin was 90%, while for distilled water, the percentage was 100%. The study also examined the effects of different filtrates on seed germination. It was observed that the number of seeds germinated after three days and the number of seedlings developed after seven days from germination ranged from 90% to 95% at higher contact times (60 and 90 min) and approximately 71% at the contact time of 30 min. These findings were consistent with the thermokinetic data, which indicated that the optimal contact time for reaching adsorption equilibrium was 60 min.

In the case of the Mn(II)-loaded Sarkanda grass lignin samples, moderate germination was observed after three days (germination energy, Eg) only at low metal concentrations (5.4938 and 10.9876 mg/L). Under these conditions, between 7 and 3 seeds out of the 10 tested germinated, depending on the contact time during the prior adsorption step (Table 4).

As the Mn(II) concentration increased to 27.469 mg/L, seed germination was severely suppressed, with only a single seed (10%) germinating at the 30-min contact time, and zero germination recorded at 60 and 90 min. For all concentration levels exceeding 32.9628 mg/L, seed germination was completely inhibited from the third day onward across all contact times, due to the high toxicity of the bound manganese. At the conclusion of the 7-day vegetation period, the germination capacity (Fg) for all Mn(II)-loaded lignin samples was 0%, regardless of the prior contact times and initial metal concentrations, as any emerged seedlings ultimately perished. This outcome strongly demonstrates the severe phytotoxic effect induced by the immobilized Mn(II) on sorghum seeds, indirectly confirming the highly efficient retention and trapping of the metal ions within the pores of the Sarkanda grass lignin.

**Figure 9:**
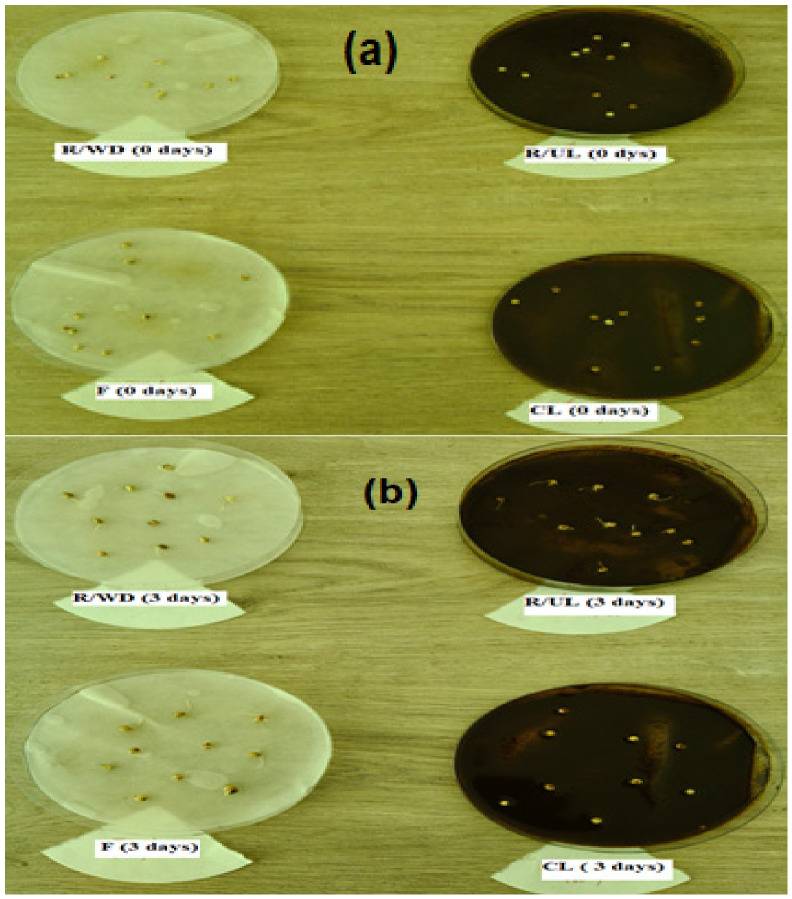

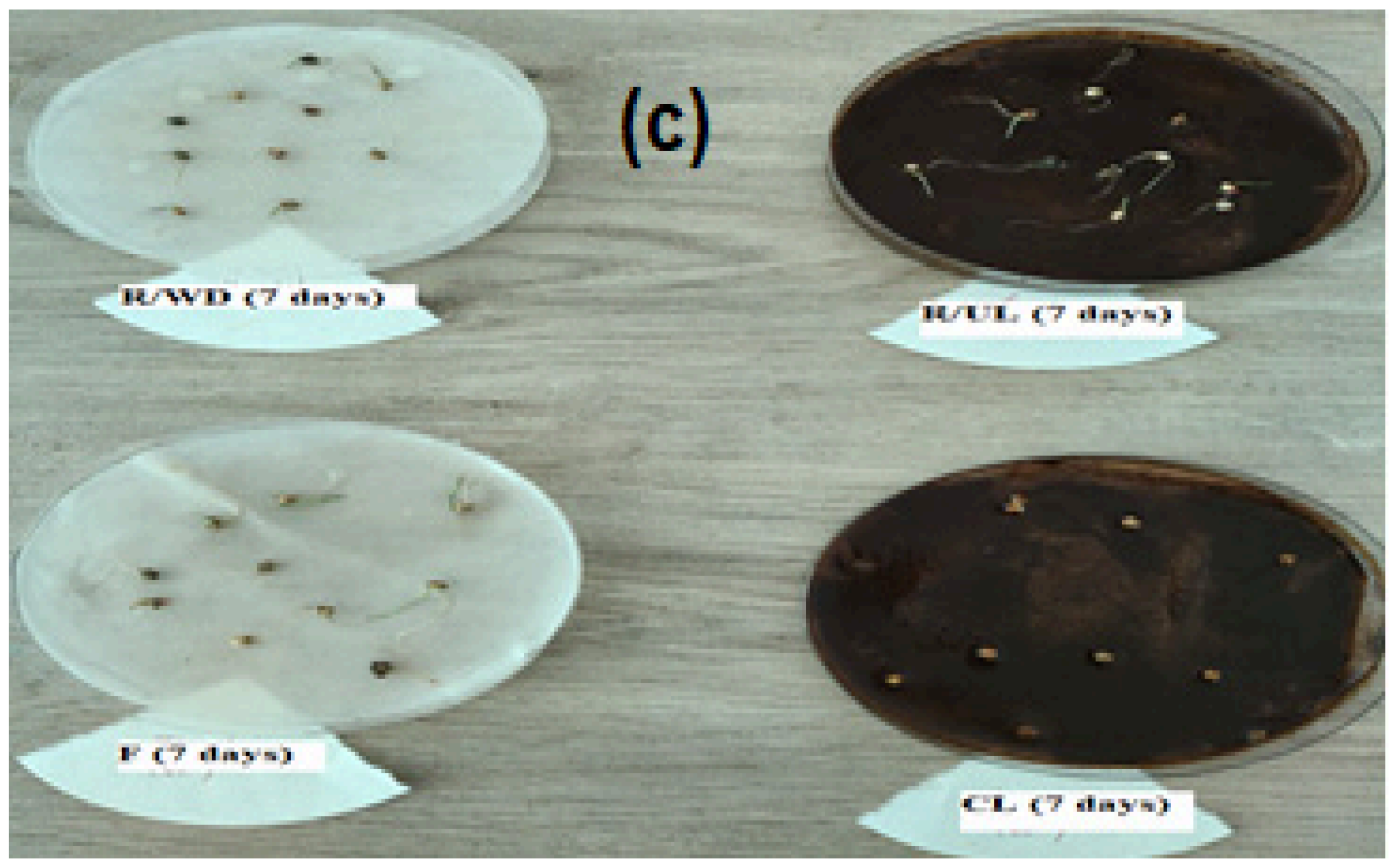
The germination progress of sorghum seeds at an adsorption time of 60 min and a concentration of 54.938 mg/L Mn(II)—after the first day of incubation (**a**), after 3 days of germination (**b**) and seedlings after 7 days of vegetation (**c**). (Note: F represent the filtrate, R/DW represent the reference in distilled water, R/UL represents the reference in uncontaminated lignin, while CL represents the lignin contaminated with a Mn(II) solution).

As seen in Figure 9, the germination of sorghum seeds is shown to occur in the presence of two distinct lignin samples: one that is uncontaminated by Mn(II) (R/UL) and another that has been contaminated with a Mn(II) solution (CL). The CL solution contains 54.938 mg of Mn(II) per litter of water. The reference/distilled water (R/DW) and the filtrate obtained after phase separation (lignin-aqueous Mn(II) 54.938 mg/L solution) were used as controls. The experiment was conducted under conditions that included a contact time of 60 min. The results obtained from this experiment serve to confirm the deductions that were previously derived from the analysis of Figure 8.

#### 3.6.2. Germination Energy and Capacity of *Sorghum bicolor* L.

As illustrated in Table 4, the values recorded for energy and germination capacity are consistent with the number of germinated seeds in the case of filtrates and lignin samples loaded with Mn(II). This indicates that although macro-kinetic equilibrium is effectively achieved at 30 min, a 60-min contact time is required operationally to ensure the absolute maturation of the lignin matrix and the complete biological detoxification of the filtrate. The evaluated biological indices exhibit higher values than their recorded values at higher contact times, i.e., 60 and 90 min, respectively. This is due to the more intense adsorption of Mn(II), which results in the filtrates being more diluted.

As illustrated in Table 4, the presence of lignin contaminated with a concentration of 32.9628 mg/L Mn(II) results in the germination energy reaching zero, while the germination capacity remains zero in all instances. The germination energy and capacity of the filtrate demonstrate variability that is analogous to that observed in the control sample, exhibiting independence from the Mn(II) concentration. These variations are statistically insignificant at the 60- and 90-min contact times, as indicated in Table 4. Consequently, while kinetic equilibrium is rapidly attained within 30 min, the 60-min interval is recommended as the optimal operational contact time to guarantee maximum ecological safety in the Sarkanda Grass-Mn(II) lignin system. This conclusion is further substantiated by biological tests that reveal the biostability of lignin [21] and, consequently, the strong affinity of the species involved in adsorption.

### 3.7. Investigation of Mn(II) Adsorption from Aqueous Solutions onto Sarkanda Grass Lignin by Morphological and Spectroscopic Analyses

#### 3.7.1. Assessment of Mn(II) Adsorption on Sarkanda Grass Lignin Using SEM–EDX Analysis

**Figure 10:**
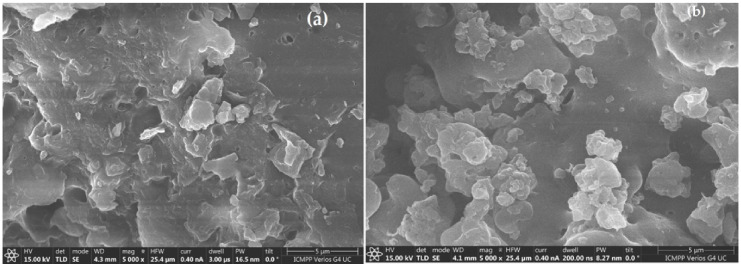
The SEM images for Sarkanda grass lignin before adsorption (**a**) and after Mn(II) adsorption (**b**).

Presently, state-of-the-art SEM-EDX technologies have attained a level of sophistication that facilitates the expeditious acquisition of comprehensive chemical information from materials, exhibiting non-destructive, quantitative, and high-resolution characteristics [83]. In light of these considerations, it was deemed beneficial to undertake these analyses for both uncontaminated and contaminated lignin with pollutant species, as well as for native sorghum seeds, respectively, for sorghum seeds that were incorporated into saturated lignin during the vegetation period (7 days). As illustrated in Figure 10a,b, the morphology of Sarkanda grass lignin is depicted before and after the retention of Mn(II) at a concentration of 54,938 mg/L and a contact time of 60 min. A comparative analysis of the micrographs (SEM) for pristine Sarkanda grass lignin (Figure 10a) and lignin treated with Mn(II) (Figure 10b) reveals some differences. The surface morphology of uncontaminated lignin typically exhibits micrometric particles with irregular shapes, while the surface morphology of contaminated lignin reveals particle agglomerations, modified appearance, and texture. These observations are indicative of the interaction between the phases involved, the diffusion and retention of Mn(II) on the lignin surface and even within its accessible pores.

**Figure 11:**
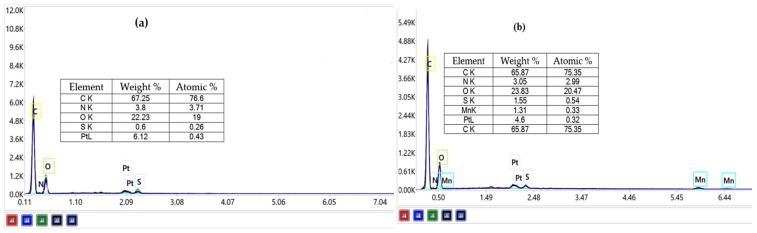
The EDX elemental analysis for lignin before adsorption (**a**) and after Mn(II) adsorption (**b**).

The (EDX) analysis unequivocally substantiates the adsorption of Mn(II) in the lignin matrix (see Figure 11a,b). A thorough examination of the EDX spectrum obtained for lignin contaminated with an aqueous solution of Mn(II) at a concentration of 54.938 mg/L, at a contact time of 60 min, and at a pH of 6.5 (see Figure 11b), reveals the presence of clear and distinct peaks corresponding to Mn(II). These peaks are not present in the spectrum of native lignin (see Figure 11a), thereby confirming the diffusion and retention of Mn(II) from the aqueous phase into the solid phase of the adsorbent.

**Figure 12:**
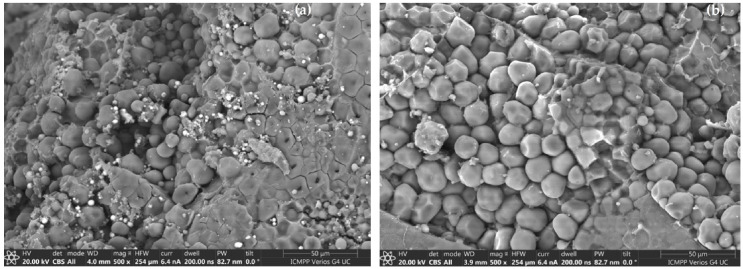
SEM images of sorghum seeds after 7 days of incubation in lignin contaminated with Mn(II) solution (54.938 mg/L, contact time: 60 min) (**a**), compared with native (uncontaminated) sorghum seeds (**b**).

As illustrated in Figure 12a, the morphology of sorghum seeds was examined after a seven-day introduction period. This investigation was conducted to assess the germination potential of the seeds in a Sarkanda grass lignin sample contaminated with a Mn(II) solution (54,938 mg/L) in an aqueous solution. The contact time for this experiment was set at 60 min. The results demonstrate that the morphology of the contaminated seeds exhibits significant alterations compared to uncontaminated sorghum seeds (Figure 12b). This observation serves as additional evidence that substantiates the retention of the metal ion within the pores of the lignin adsorbent.

**Figure 13:**
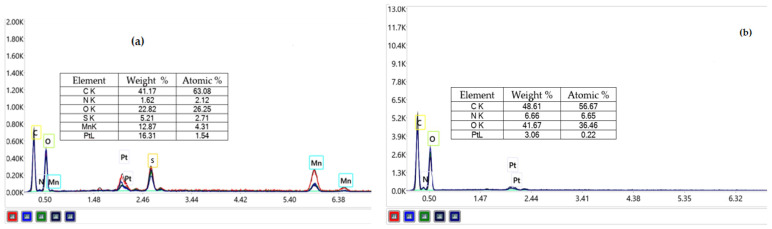
The EDX elemental analysis for sorghum seeds embedded for 7 days in lignin contaminated with Mn(II) solution 54.938 mg/L, contact time 60 min (**a**) and for native (uncontaminated) sorghum seeds (**b**).

As illustrated in Figure 13a,b, the EDX spectra for sorghum seeds that were incorporated during the vegetation period (7 days) were evaluated to ascertain the germination capacity in Sarkanda grass lignin contaminated with Mn(II) 54.938 mg/L aqueous solution, contact time 60 min (Figure 13a), respectively, for uncontaminated sorghum seeds (Figure 13b).

A comparison of the two spectra reveals clear differentiations that substantiate the retention of Mn(II) in the pores of Sarkanda grass lignin. This assertion is further substantiated by the presence of peaks corresponding to Mn(II) in the spectrum of seeds introduced into the contaminated lignin and, by extension, in the contaminated seeds (see Figure 13a). Notably, these peaks are absent from the spectrum of native sorghum seeds (see Figure 13).

#### 3.7.2. Confirmation of Mn(II) Adsorption on Sarkanda Grass Lignin Using FTIR Spectroscopy

**Figure 14:**
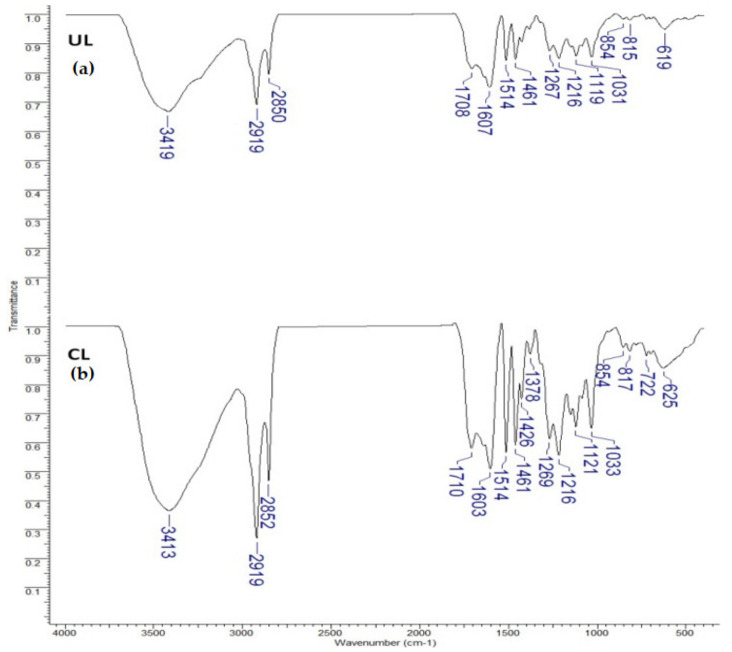
FTIR spectra for Sarkanda grass lignin before adsorption (**a**) and after Mn(II) adsorption (**b**), contact time 60 min.

Given that FTIR spectroscopy is an analytical technique of high accuracy and sensitivity [84], providing structural and functional information extremely useful for the detection and characterization of complex systems [85], its use has become particularly frequent. As illustrated in Figure 14a,b, the FTIR spectra for unmodified lignin (UL) and lignin contaminated with an aqueous solution of Mn(II) at a concentration of 54.938 mg/L and a contact time of 60 min (CL) are presented, respectively.

The unmodified lignin (UL) exhibits broad O–H stretching bands at 3419 cm^−1^, attributed to hydroxyl groups in phenolic and aliphatic structures. Peaks at 2919 and 2850 cm^−1^ correspond to C–H stretching in aromatic methoxyl groups and methyl/methylene side chains or fatty acids present in lignin. The band at 1607 cm^−1^ is related to protein impurities and water associated with lignin. Absorptions at 1514, 1267, 1119, and 815 cm^−1^ are assigned to C–H deformation, guaiacyl (G) unit ring vibrations and C = O stretching, C–H in-plane bending, and C–H out-of-plane bending at positions 2, 5, and 6 of guaiacyl units [56]. The spectrum of lignin contaminated with Mn(II) shows peaks specific to the two precursors, but some differences also appear.

The kinetic modeling and thermodynamic data suggest a chemisorption process, but a deeper understanding of the binding mechanism requires elucidation of the specific coordination pathways on the lignin surface. Initially, at the optimal pH of 6.5, the highly deprotonated carboxyl groups on the Sarkanda grass lignin create a strong negative electrostatic field, driving the rapid migration of solvated Mn^2+^ ions from the bulk solution to the solid-liquid interface.

Following this initial electrostatic attraction, the adsorption transitions into a chemically driven inner-sphere complexation. As detailed in recent advanced studies on biopolymer-metal coordination [86], transition metals like Mn^2+^ exhibit a preferential coordination mechanism. The electron-rich oxygen atoms within the lignin matrix—specifically the deprotonated carboxyl groups, phenolic hydroxyls and adjacent methoxy groups characteristic of lignin units—act as strong Lewis donor sites for metal coordination. The Mn^2+^ ion, acting as a Lewis acid, preferentially coordinates with these adjacent oxygen-containing sites, displacing hydration water molecules to form stable, multi-ring chelate structures.

This localized preferential coordination is directly corroborated by our FTIR findings, where the significant shifts in the O–H (3400 cm^−1^) and aromatic C–O (1200 and 807 cm^−1^) vibrational bands indicate that the oxygen atoms in the phenolic structures are actively sharing electron density with the manganese ions, forming strong, dative covalent bonds. Thus, the overall high removal efficiency of the lignin is not merely a consequence of passive electrostatic trapping, but rather the result of a highly specific and thermodynamically stable preferential coordination framework.

**Figure 15:**
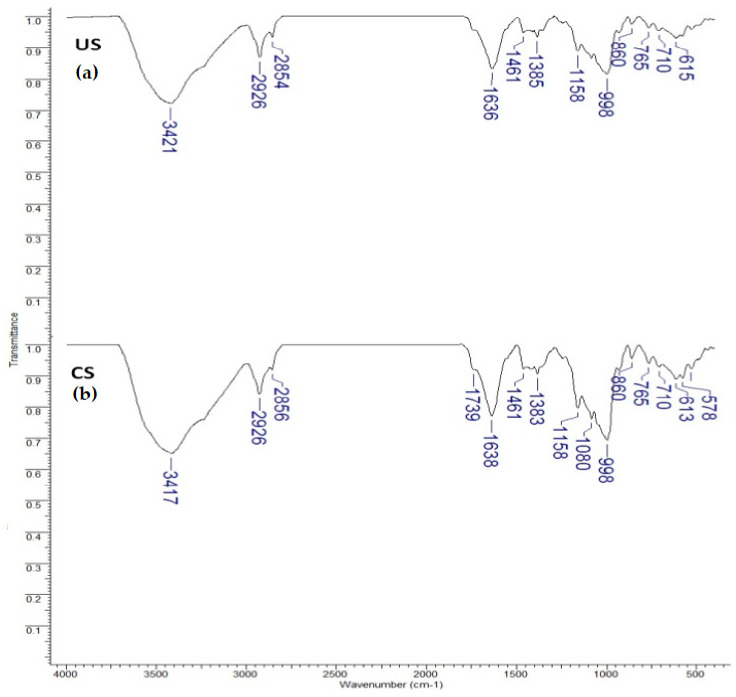
FTIR spectra for native (uncontaminated) sorghum seeds (**a**) and sorghum seeds embedded for 7 days in lignin contaminated with Mn(II) solution 54.938 mg/L, contact time 60 min (**b**).

The FTIR spectra for uncontaminated sorghum seeds (US) and for the sorghum seeds that were incorporated for seven days into lignin contaminated with a solution of Mn(II) at a concentration of 54.938 mg/L (treated sorghum seeds (CS)) are shown in Figure 15a,b.

The spectrum for uncontaminated sorghum seeds (US) shows a strong, broad absorption band representing bonded O-H stretching (indicating moisture, starches, and cellulose) on 3421 cm^−1^. At 2926 and 2854 cm^−1^: symmetrical and asymmetrical C-H stretching from lipids, starches, and proteins. The band at 1636 cm^−1^ is related to amide I associated with the secondary structure of kafirins (sorghum proteins) and absorbed water. On 1158 cm^−1^: C-O and C-C stretching vibrations indicative of the saccharide rings in starch [56].

The contaminated samples (CL, CS) show changes in the infrared spectrum. In the case of lignin, the bands from 1119 and 1031 cm^−1^ (C–H deformation) moves to 1121 and 1033 cm^−1^ and are more prominent indicating the presence of sulfate groups (SO_4_^2−^: asymmetric stretching ν3). The peak at 625 cm^−1^ indicates metal-oxygen lattice vibrations (manganese ion), thus confirming the achievement of adsorption. The same aspect can be observed in the CS sample, small wavenumber shifts and more prominent bands in the case of sulfates attached to sorghum and the presence of manganese ion at 578 cm^−1^.

**Figure 16:**
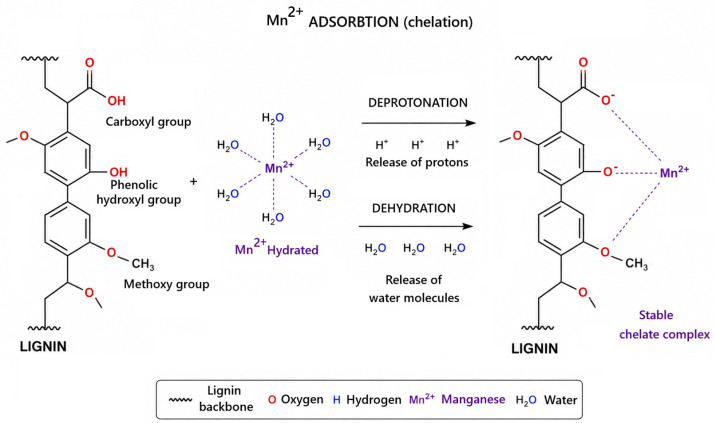
Schematic representation of the proposed Mn(II) adsorption mechanism on Sarkanda grass lignin.

These changes indicate the existence of chemical interactions between lignin and Mn(II), thereby confirming the occurrence of adsorption. Based on cummulative spectroscopic evidence (FTIR and EDX) and the thermodynamic data, the proposed adsorption mechanism of Mn(II) onto Sarkanda grass lignin is illustrated in Figure 16. The mechanism proceeds in three distinct stages: (i) electrostatic attraction between the Mn^2+^ ions and the deprotonated carboxyl/phenolic groups at pH 6.5; (ii) the dehydration of the hydrated [Mn(H_2_O)_6_]^2 +^ ions, which accounts for the observed entropy increase; and (iii) the formation of inner-sphere coordinate bonds (chelation) between the metal ion and the oxygen-containing functional groups (–COO^−^, –O^−^, and –OCH_3_) of the lignin matrix, resulting in a stable multi-ring chelate structure.

### 3.8. Assessment of Mn(II) Adsorption Efficiency on Spent and Regenerated Sarkanda Grass Lignin over Three Cycles Based on Retention Percentage

For an adsorbent to be considered viable in industrial environments, its capacity to recover and reuse after adsorption is of paramount importance, particularly within the framework of a circular bioeconomy. Therefore, in order to evaluate the stability and recycling potential of used Sarkanda grass lignin, a series of three systematic adsorption-desorption tests was conducted. The adsorbent was regenerated with 1N HCl [21]. The experimental data obtained (Table 5) indicate a potential for reuse without significant losses in adsorption efficiency. As demonstrated in Table 5, the adsorption rate for the regenerated lignin exhibited a high stability for the metal ion, with minor fluctuations during the regeneration cycles, primarily attributable to lignin pore clogging.

Therefore, for a contact time of 60 min, which is regarded as the optimal operational contact time, a retention ranging from 83 to 89% is observed after the initial cycle, with an average of 85.8%. Subsequent to the second cycle, a retention ranging from 76 to 82% is observed, with an average of 79.4%. Following the third cycle, a retention ranging from 67 to 73% is observed, with an average of 70.7%. At a contact time of 90 min, effective retention percentages are documented as well. In the initial cycle, the recorded percentages range from 83–89%, with an average of 85.1%. In the subsequent cycle, the range is from 76–82%, with an average of 79.8%. Finally, in the third cycle, the range is from 68–74%, with an average of 71%.

As indicated in Table 5, the initial contact time (30 min) exhibited lower retention percentages compared to the subsequent two contact times. However, it is noteworthy that the average retention percentages for each cycle consistently surpassed 50%, underscoring the efficacy of the experimental approach. Therefore, following the initial retention cycle, percentage values ranging from 68 to 74% are obtained, with an average of 71.2%. Subsequent to the second cycle, values between 59 and 66% are recorded, with an average of 63.2%.

Finally, after the third cycle, the values obtained are between 53 and 59%, with an average of 56%. The experimental data obtained suggest that a duration of 60 min is the optimal operational time to ensure maximum process efficiency and complete maturation of the lignocomplexes.

Although the retention capacity remains viable for practical applications, the observed decrease from an average of 85.8% in the first cycle to 70.7% in the third cycle warrants an in-depth mechanistic analysis. This attenuation in adsorption efficiency is not merely a consequence of physical exhaustion, but rather the result of several synergistic physicochemical factors acting upon the lignin matrix.

First, the structural integrity of the lignin is gradually altered by the regeneration process itself. Repeated exposure to a strong acidic medium (1N HCl) can induce structural modifications within the biopolymer. Acidic environments promote partial acid-catalyzed condensation reactions within the lignin macromolecule (leading to the formation of rigid carbon-carbon linkages), which ultimately contracts the polymer network and reduces the accessible surface area [87]. Furthermore, the strong acid causes persistent protonation of the highly active carboxyl and phenolic hydroxyl groups, thereby diminishing the net negative surface charge required for optimal electrostatic attraction in subsequent cycles [88].

Second, the decrease is intensified by incomplete desorption. Because the adsorption of Mn(II) is driven by robust inner-sphere complexation (strong dative bonds) rather than weak physical trapping, the acidic eluent cannot completely cleave all the established Mn–O coordinate bonds. Consequently, a fraction of the heavy metal remains irreversibly bound, leading to the progressive saturation and steric blockage of active chelating sites [89].

Finally, from a macroscopic operational standpoint, the repeated cycles of mechanical stirring, acidic washing, and filtration inevitably result in the minor loss of fine adsorbent particles, which cumulatively lowers the overall available mass of the lignin fraction. Together, these structural, chemical, and physical factors account for the gradual decline in retention efficiency over multiple cycles [90].

Besides al of these, lignin can be regarded as a promising sustainable alternative for removing pollutants, such as heavy metal ions (e.g., Mn(II)) from aqueous environments. This finding supports the continuation of investigations on the regeneration processes of the biomass fraction. The findings obtained from this study are consistent with the available literature concerning the retention percentage of heavy metal ions on regenerated low-cost adsorbents. The present literature attributes an efficiency of approximately 80% after three or even five adsorption-desorption cycles [91], with minor discrepancies depending on the adsorbed metal ion. However, these discrepancies are statistically insignificant [18,58,92].

At the optimal operational contact time (60 min), the retention percentage of Mn(II) from an aqueous medium after three cycles of Sarkanda grass lignin regeneration ranges from 70.7% to 85.8% (see Table 5). A review of the pertinent literature reveals that, following a polycyclic regeneration, the retention percentages of heavy metals on low-cost adsorbents are notably elevated. For instance, the retention percentage for chitosan ranges from 75% to 90% [93], for cellulose it ranges from 65% to 70% [58,94], for walnut shells it ranges from approximately 85% [95], for corn pan it ranges from approximately 70% [96], for natural zeolites (primarily clinoptilolite) it ranges from 70% to 95% [16], and for sawdust it ranges from 80% to 95% [97]. Consequently, the regenerated Sarkanda grass lignin is found within the documented value ranges of other regenerated bio-sorbents.

### 3.9. Comparative Evaluation of Mn(II) Retention Efficiency of Sarkanda Grass Lignin and Other Biomass Fractions in Aqueous Media

The industrial scalability of *Tripidium bengalense* (Sarkanda grass) lignin is strongly supported by its established role as a secondary raw material in the pulp and paper industry. As demonstrated by Sarkanda grass is characterized by its high abundance and low-lignin content, allowing for efficient pulping under mild conditions compared to woody feedstocks [98]. This facilitates a ‘cascading’ industrial utilization, where the plant is processed for fiber (for writing and printing-grade paper), while the resulting lignin-rich byproduct is diverted for high-value environmental remediation98].

Structurally, *Tripidium bengalense* fibers possess a significant chemical potential, with a cellulose content of approximately 53.45% and a balanced composition that contributes to a high crystallinity index and thermal stability [99]. From the perspective of lignin chemistry, the ‘technical lignin’ obtained as a byproduct of these industrial processes is particularly advantageous for adsorption applications. Unlike certain Kraft lignins that are heavily condensed, the soda-process lignin from Sarkanda grass exhibits specific molar mass distributions and chemical compositions that render it highly effective for chemical functionalization and binding applications. Research comparing industrial lignins has identified that Sarkanda grass soda lignin fractions, particularly those with medium molar mass, possess significant potential for chemical industry applications due to their reactive sites, which are essential for metal complexation [100].

The amorphous three-dimensional structure of lignin allows for its transformation and optimization, thereby enhancing its adsorption capacity and selectivity [21]. Conversely, there are also other abundant biomass fractions (e.g., cellulose, hemicellulose, chitosan, alginates, pectins) [101] with a predisposition to adsorption processes due to different functional groups (aromatic, phenolic hydroxyl, alcoholic hydroxyl, carbonyl, methoxy, carboxyl, and amino groups, conjugated double bond), which have the capacity to form chemical bonds with metal ions [92].

However, the structural recalcitrance and crystallinity of these biopolymers dictate their binding efficiency. For instance, cellulose—the most abundant biopolymer in nature—is characterized by a highly ordered semi-crystalline structure. Despite possessing a high theoretical density of hydroxyl groups, strong intra- and intermolecular hydrogen bonding renders these functional groups largely inaccessible to solvated metal ions. Consequently, raw cellulose exhibits significantly fewer active binding sites for metals compared to lignin. Lignin’s chaotic, amorphous network provides an extended, highly accessible surface area, rich in phenolic hydroxyl and carboxyl groups (resulting from extraction processes), which act as powerful electron donors for rapid and efficient transition metal chelation [101].

To accurately position Sarkanda grass lignin within the broader context of eco-friendly adsorbents, Table 6 presents a comparative evaluation of Mn(II) adsorption capacities across various raw and modified biomass fractions reported in recent literature. Chitosan exhibits a comparable performance to lignin, acting as a powerful chelating agent due to its abundant amine and hydroxyl groups, achieving retention percentages exceeding 90% [102]. This affinity recommends its future utilization in synergistic combination with lignin to develop advanced biocomposites or hybrid materials and requires continued research in order to design adsorption systems optimized by experimental conditions and personalized on the speciation of the polluting metal ion [103].

As evidenced in Table 6, untreated agricultural residues and native biomass fractions frequently exhibit limited adsorption capabilities for Mn(II). For instance, raw banana peel biochar (0.80 mg/g), raw Moringa oleifera seed pods (6.00 mg/g), and coffee pulp (8.01 mg/g) demonstrate significantly lower affinities for manganese ions. In this context, the untreated Sarkanda grass lignin evaluated in the present study demonstrates a highly competitive retention capacity of 12.52 mg/g, outperforming several common raw biosorbents and remaining comparable to raw coconut shell powder (13.90 mg/g).

A critical analysis of the literature reveals that extraordinarily high adsorption capacities (q_max_ > 30 mg/g) are almost exclusively achieved through severe chemical modifications. For example, the oxidation of cellulose using TEMPO or sodium periodate (NaIO_4_) drastically breaks the crystalline structure and introduces massive amounts of highly reactive carboxyl and aldehyde groups, boosting the capacity to 52.90 mg/g and 32.20 mg/g, respectively. Similarly, treating rice husk with nitric acid (HNO_3_) raises its capacity to 36.17 mg/g by artificially increasing surface oxygen-containing functional groups. However, from the perspective of sustainability and the circular bioeconomy, these chemical modifications present significant drawbacks. The use of strong acids (HNO_3_, H_3_PO_4_), complex oxidants (TEMPO), or thermal activation processes requires substantial energy input and generates toxic secondary effluents, essentially conflicting with the “zero waste” philosophy.

It is particularly noteworthy that, while the biosorption of highly toxic heavy metals (e.g., Pb(II), Cu(II), Cd(II), and Cr(VI)) by various technical lignins has been extensively documented, there remains a conspicuous research gap regarding the remediation of manganese [114,115]. Despite being a ubiquitous and hazardous contaminant in industrial and mining wastewaters, an exhaustive literature review reveals a striking absence of studies evaluating unmodified, raw lignins specifically devoted to its removal. Consequently, a direct quantitative comparison between Sarkanda grass lignin and other native wood- or grass-derived lignins for Mn(II) adsorption is currently constrained by the unavailability of published data.

This pronounced scarcity in the literature elevates the scientific novelty of the present study. By comprehensively characterizing the Mn(II) retention capacity of unmodified Sarkanda grass lignin (12.52 mg/g), this work establishes a pioneering benchmark. It provides critical foundational data regarding the affinity of herbaceous lignin’s carboxyl and phenolic hydroxyl groups for Mn(II) chelation, serving as a vital reference point for future comparative studies involving diverse industrial lignin fractions.

In this context, the present research recommends lignin from Sarkanda grass as an efficient biosorption agent for the retention of Mn(II) under static conditions in aqueous environments. This recommendation is based on the reproducibility of the experimental results (morphological, compositional, and spectral observations combined with chemical equilibrium data, thermokinetics, thermostability, and biostability).

Furthermore, the material’s robust performance in both its native and regenerated states, with effective retention maintained over consecutive cycles, provides a scalable foundation for practical field applications. Moving forward, research priorities should transition from static batch-scale configurations toward continuous-flow systems and the design of hybrid biocomposite architectures. Such advancements will ensure that lignin is fully harnessed as a resilient, natural ‘barrier’ to heavy metal contamination, ultimately maximizing its potential within the global sustainable remediation landscape.

The technical profile of Sarkanda lignin establishes it as more than a ordinary laboratory-scale biosorbent; it is an industrially sourced byproduct with an existing, scalable supply chain. By redirecting this material from pulp and paper waste streams toward Mn(II) sequestration, our approach not only upholds the core tenets of the circular bioeconomy but also offers a significant reduction in the carbon footprint typically associated with the synthesis of specialized, petroleum-derived adsorbents.

## 4. Conclusions

Lignin, a renewable and highly available resource, is an aromatic biopolymer with a complex structure. It has been shown to have a high abundance and variety of functional groups that are active in deprotonation and chelation with heavy metal ions [116,117]. These attributes recommend it for potential biosorption functions [118].

In this study, the efficience of Sarkanda grass lignin as an adsorbent for Mn(II) from aqueous media is examined under meticulous experimental conditions. The temperature is maintained at 20 ± 0.1 °C, and the pH is adjusted to 6.5 for both lignin and the aqueous solutions of the contaminant species. The adsorbent dosage is set at 5 g/L of the Mn(II) solution, and the concentration range is varied from 5.4938 to 54.938 mg/L of Mn(II). The contact time, maintained under static conditions, is set to 60 min between the phases involved. The evaluation of the performance of Mn(II) adsorption from aqueous media onto Sarkanda grass lignin was based on several types of analyses. These analyses were conducted in order to verify the reproducibility of the experimental data. The purpose of this was to draw a conclusion regarding the possible usefulness of the bioresource as a biosorbent.

Therefore, the R^2^ correlation coefficients responsible for validating the performance of an adsorbent from an application point of view, obtained based on the experimental Freundlich and Langmuir adsorption isotherms, indicate the probability of a chemical adsorption carried out in the polylayer. Consequently, the Freundlich model fits better in describing the retention of Mn(II) from aqueous media on Sarkanda grass lignin. The experimental isotherms were interpreted, and the resulting deductions were found to be in full agreement with the adsorption thermodynamics of the investigated system. The calculated thermodynamic indices indicated a possible endothermic, spontaneous, and partially disordered character, as evidenced by a decrease in Gibbs free energy and an increase in entropy and enthalpy. This finding suggests a thermodynamic compartment favorable to adsorption, thereby demonstrating the feasibility of the process [119]. This phenomenon has been previously observed in adsorbents that have been validated [120].

The kinetics of Mn(II) adsorption from aqueous media onto Sarkanda grass lignin is more accurately reproduced by the pseudo-II order Ho-McKay model than by the pseudo-I order Lagergren model. This is evidenced by the values of the R^2^ correlation coefficients being unity in all cases, indicating the probability of electrostatic interactions [117] between the species involved. This finding is consistent with the conclusion offered by the Freundlich model, which supports the probability of a chemisorption attributed to the appearance of the dative bond that favors the formation of relatively stable chelates [117] and contributes to the feasibility of the process [121].

Thermal analyses (TG-DTG) demonstrate the efficiency of Sarkanda grass lignin as an adsorbent for Mn(II) from aqueous media, through the difference recorded in the mass residues, in this case 37.11% for uncontaminated lignin and 52.94% for lignin contaminated with Mn(II) 54.938 mg/L.

The adsorption capacity of Sarkanda grass lignin for manganese (II) from aqueous solutions was verified through the implementation of biological tests on *Sorghum bicolor* L. seeds. These seeds were introduced for a period of seven days into lignin that had been contaminated with metallic species at concentrations ranging from: The concentration of the substance in question was measured to be between 5.4938 and 54.938 mg/L. This measurement was obtained from the filtrates that were obtained subsequent to the isolation of the post-adsorption phases, and this measurement was taken at the three contact times. In order to obtain validation from an ecological point of view, the duration of the study should be 30, 60, or 90 min. Therefore, the results of the germination tests and the evaluated biological indices (germination energy and germination faculty) are indicative of the presence of detoxified filtrates and, consequently, the effective retention of Mn(II) in the pores of Sarkanda grass lignin. This is evident from the toxicity of contaminated lignin, which has been shown to create an unfavorable environment for the biological processes of plant systems.

SEM, EDX, and FTIR analyses were performed on both uncontaminated samples (lignin and sorghum seeds) and on lignin and plant samples treated with the polluting species. The analyses unanimously confirmed the retention of Mn(II) in the lignin matrix and the post-adsorption morphological changes on the surface of Sarkanda grass lignin and sorghum seeds. These changes transform lignin into a sustainable alternative in the transition to a circular economy and bioeconomy. Furthermore, the probability of generating a closed-loop economic system that induces scalability was determined.

Furthermore, the regeneration experiments conducted on saturated Sarkanda grass lignin demonstrated the efficacy of the reused adsorbent following polycycles of adsorption-desorption. At the optimal contact time (60 min), a retention of over 70% (70.7–85.8%) was recorded, aligning with the findings reported in other low-cost adsorbents [91]. This propels lignin into an exceptionally valuable bioresource for circular economy models, which prioritize regenerability, biodegradability, profitability, relatively facile processing, biocompatibility, revalorization, and “zero waste” generation [21]. This biomass fraction exhibits attributes that converge towards sustainability, yet its full potential remains untapped and the present study proposes to provide an additional argument regarding the suitability of transforming lignin from an agro-industrial by-product into a scalable element.

## Figures and Tables

**Table 1:** Characteristic parameters of Freundlich and Langmuir models for Mn(II) adsorption on Sarkanda grass lignin.

Pollutant	Time (min)	Freundlich Model			Langmuir Model		
		R^2^	1/*n*	k_F_	R^2^	q_m_ (mg/g)	K_L_
Mn(II)	30	0.9886	0.9002	1.8953	0.9091	12.2021	0.0815
	60	0.9921	0.9358	2.2188	0.8603	12.3762	0.0805
	90	0.9891	0.9973	2.0736	0.7536	12.3824	0.0804

**Table 2:** Thermodynamic parameters obtained for the adsorption of Mn(II) ions from aqueous solutions onto Sarkanda grass lignin.

Pollutant	pH	Time (min)	ΔG at 20 °C (293.15 K) (kJ/mol)	ΔG at 25 °C (298.15 K) (kJ/mol)	ΔG at 30 °C (303.15 K) (kJ/mol)	ΔH (kJ/mol)	ΔS (J/mol K)
Mn(II)		30	−14.53	−15.00	−15.47	12.98	93.84
	2.64	60	−13.78	−14.20	−14.63	11.21	85.24
		90	−13.24	−13.68	−14.13	12.77	88.73
		30	−14.85	−15.35	−15.86	14.83	101.23
	6.5	60	−24.89	−25.54	−26.19	13.21	129.96
		90	−20.35	−20.94	−21.52	14.02	117.24

**Table 3:** Kinetic parameters of the Lagergren and Ho-McKay models for Mn(II) adsorption on Sarkanda grass lignin.

Pollutant	c_i_ (mg/L)	Lagergren Model			Ho-McKay Model		
		R^2^	q_e_ (mg/g)	K_1_ (min^−1^)	R^2^	q_e_ (mg/g)	K_2_ (g/mg·min)
Mn(II)	10	0.7231	1.1056 (±0.12)	−0.0017	1	0.9218 (±0.06)	1.0123
	20	0.7112	1.1448 (±0.09)	−0.0014	0.9999	5.3706 (±0.11)	2.1238
	30	0.7495	4.1713 (±0.18)	−0.0015	1	3.2397 (±0.09)	4.0006
	40	0.5978	4.3927 (±0.08)	−0.0019	0.9998	5.9782 (±0.07)	2.2273
	50	0.6827	5.4113 (±0.17)	−0.0017	1	7.7657 (±0.14)	2.7598
	60	0.8358	5.9197 (±0.05)	−0.0015	1	8.6706 (±0.08)	2.2793
	70	0.8211	7.0242 (±0.14)	−0.0018	1	9.6847 (±0.16)	1.6578
	80	0.7402	7.6231 (±0.16)	−0.0018	1	9.8904 (±0.11)	4.5862
	90	0.7475	7.8285 (±0.21)	−0.0016	1	10.6009 (±0.19)	2.6185
	100	0.8516	8.1093 (±0.07)	−0.0017	1	10.4493 (±0.13)	2.3136

**Table 4:** Mean germination energy and capacity, determined in triplicate, for the contaminated samples and for the filtrates obtained after Mn(II) retention at the three experimental contact times.

Lignin/Mn(II) (mg/L)	Contact Time (min)						Lignin/Mn(II) (mg/L) Filtered	Contact Time (min)					
	30	60	90	30	60	90		30	60	90	30	60	90
	Eg, %			Fg, %				Eg, %			Fg, %		
0	90	90	90	90	90	90	0	100	100	100	100	100	100
5.4938	70	60	60	0	0	0	5.4938	80	90	80	80	90	90
10.9876	50	50	30	0	0	0	10.9876	80	90	90	90	90	100
16.4814	40	30	30	0	0	0	16.4814	70	100	90	90	100	100
21.9752	20	10	10	0	0	0	21.9752	70	100	100	90	100	100
27.469	10	0	0	0	0	0	27.469	70	90	90	80	90	90
32.9628	0	0	0	0	0	0	32.9628	60	100	100	70	100	100
38.4566	0	0	0	0	0	0	38.4566	60	100	100	60	100	100
43.9504	0	0	0	0	0	0	43.9504	50	90	90	50	90	100
49.4442	0	0	0	0	0	0	49.4442	50	90	90	50	90	90
54.938	0	0	0	0	0	0	54.938	50	100	90	50	100	90

**Table 5:** Retention Percentage (R) of Mn(II) on Spent Sarkanda grass Lignin during Three Desorption–Readsorption Cycles at the Experimental Contact Times.

Lignin/Mn(II) (mg/L)	Contact Time (Min)								
	30	60	90	30	60	90	30	60	90
	First Cycle			Second Cycle			Third Cycle		
	R, %			R, %			R, %		
5.4938	68	83	83	59	76	76	53	67	68
10.9876	69	84	84	61	77	77	54	68	68
16.4814	69	84	84	61	78	78	54	69	69
21.9752	70	85	85	62	79	79	55	70	70
27.469	71	85	86	64	79	80	55	71	71
32.9628	72	86	86	64	80	80	56	71	72
38.4566	72	87	87	65	80	81	57	72	72
43.9504	73	87	88	65	81	82	58	73	73
49.4442	74	88	89	65	82	82	59	73	73
54.938	74	89	89	66	82	82	59	73	74
Median	71.2	85.8	86.1	63.2	79.4	79.8	56	70.7	71

**Table 6:** Comparison of maximum Mn(II) adsorption capacities (q_max_) of various untreated and modified biomass-derived adsorbents.

Adsorbent Material	Modification/Treatment	pH	q_max_, mg/g	Reference
Sarkanda grass lignin	Untreated (Raw)	6.5	12.52	This study
Banana peels biochar	Untreated (Raw)	7	0.8	[104]
	Oxidized with H_3_PO_4_	7	23	[104]
Rice straw biochar	Unmodified	6	20.59	[105]
	Pre-alkali	6	28.37	[105]
	Post alkali	6	8.06	[105]
Rice husk	Oxidized with HNO_3_	7	36.17	[106]
Coffee pulp	Untreated (Raw)	4	8.01	[107]
*Moringa oleifera* seed pods	Untreated (Raw)	6	6	[108]
	Alkali treated	6	7.64	[108]
Cellulose	Oxidized with NaIO_4_	9	32.2	[109]
Cellulose	TEMPO oxidation	6.5	52.9	[110]
Activated carbon	Sugarcane bagasse + H_3_PO_4_	6.3	5.40	[111]
Coconut shell powder	Untreated (Raw)	7	13.9	[112]
Lignite	Untreated (Raw)	6	25.84	[113]

## Data Availability

The original contributions presented in this study are included in the article. Further inquiries can be directed to the corresponding authors.
